# Fatigue, Sleep, and Autoimmune and Related Disorders

**DOI:** 10.3389/fimmu.2019.01827

**Published:** 2019-08-06

**Authors:** Mark R. Zielinski, David M. Systrom, Noel R. Rose

**Affiliations:** ^1^Veterans Affairs Boston Healthcare System, Boston, MA, United States; ^2^Department of Psychiatry, Harvard Medical School, Boston, MA, United States; ^3^Department of Medicine, Harvard Medical School, Boston, MA, United States; ^4^Department of Pulmonary and Critical Care Medicine, Brigham and Women's Hospital, Boston, MA, United States; ^5^Department of Pathology, Brigham and Women's Hospital, Harvard Medical School, Boston, MA, United States

**Keywords:** autoimmune, cytokines, fatigue, inflammasome, inflammation, sleep, neurovascular unit

## Abstract

Profound and debilitating fatigue is the most common complaint reported among individuals with autoimmune disease, such as systemic lupus erythematosus, multiple sclerosis, type 1 diabetes, celiac disease, chronic fatigue syndrome, and rheumatoid arthritis. Fatigue is multi-faceted and broadly defined, which makes understanding the cause of its manifestations especially difficult in conditions with diverse pathology including autoimmune diseases. In general, fatigue is defined by debilitating periods of exhaustion that interfere with normal activities. The severity and duration of fatigue episodes vary, but fatigue can cause difficulty for even simple tasks like climbing stairs or crossing the room. The exact mechanisms of fatigue are not well-understood, perhaps due to its broad definition. Nevertheless, physiological processes known to play a role in fatigue include oxygen/nutrient supply, metabolism, mood, motivation, and sleepiness—all which are affected by inflammation. Additionally, an important contributing element to fatigue is the central nervous system—a region impacted either directly or indirectly in numerous autoimmune and related disorders. This review describes how inflammation and the central nervous system contribute to fatigue and suggests potential mechanisms involved in fatigue that are likely exhibited in autoimmune and related diseases.

## Introduction

According to the National Institutes of Health, autoimmune diseases are estimated to afflict over 20 million individuals in the United States (1, 2). Currently, there are over 100 recognized autoimmune diseases (3), and the prevalence of many autoimmune diseases continues to rise (4, 5). A recent self-reported survey of individuals with autoimmune and related disorders by the American Autoimmune and Related Disorders Association indicated that this population's primary concern is fatigue (6). Over two-thirds of respondents reported that their fatigue was profound, debilitating, and prevented them from completing simple everyday tasks. Indeed, a growing literature indicates that fatigue is common in most autoimmune-related diseases, as well as among individuals with related immunodeficiency disorders (7–12). Furthermore, it is estimated that 7–45% of people in the general population exhibit persistent fatigue (13), while almost 98% of individuals with autoimmune disease report that they suffer from fatigue (6). Fatigue can cause dramatic impairments in mood (14), diminish social aspects of life (15), lead to an inability to perform routine daily activities (16), and limit physical activity and work (17). Consequently, fatigue can severely affect well-being and has a financial burden on the individual, family, and society (18–21).

Fatigue is multifaceted and typically broadly defined making it difficult to decipher the causes in specific autoimmune diseases (19). Fatigue is generally described as a condition with prolonged periods of exhaustion accompanied by the inability to perform activities to an expected capacity. There are numerous aspects of fatigue that can be assessed that define the type of fatigue including the severity of functional impairment; time-of-day/circadian patterns of fatigue; length of the persistence of the fatigue from seconds to days; time between fatigue periods; duration of time necessary for the fatigue to dissipate; influence of sleep loss or disturbances in sleep; impact of depression or anxiety; degree of distress concurrently occurring with the fatigue; type of impairment, such as is cognition, motivation, attention, or physical abilities; and the type of physical performance or activity that is impaired, such as in walking, climbing stairs, socialization, chores, cooking, bathing, work, and sex. The type of fatigue experienced in autoimmune disease is variable These differences are likely related to the particular tissues/organs, cell types, brain areas, and molecular and physiological mechanisms affected by the condition (19).

Currently, there is a lack of efficacious long-lasting treatments for individuals experiencing fatigue in autoimmune disease. This is due, in part, to the limitations in our understanding of the multiple mechanisms responsible for fatigue. Evidence suggests several physiological functions can contribute to fatigue including oxygen/nutrient supply (22, 23), metabolism (24), mood (14), motivation (25), and daytime sleepiness (26, 27). Interestingly, inflammation is altered by many of the factors that modulate fatigue and vice versa (25, 28–30). Growing evidence indicates that neuroinflammation is a primary factor contributing to fatigue (25, 31). Since inflammation plays a large part in inducing fatigue, it is plausible that inflammatory pathways and the subsequent physiological alterations modulated by the inflammation are treatable targets for fatigue in patients with autoimmune disease. Indeed, evidence in autoimmune and related conditions, such as neurosarcoidosis, which is associated with increased lung inflammation, sleep disturbances, and fatigue, exhibit reduced fatigue from anti-inflammatory treatment (32). Herein, we discuss the role of factors contributing to fatigue in autoimmune disease including inflammatory-related mechanisms, relationships between peripheral and central nervous system (CNS) inflammation, particular brain areas and neurotransmitters, and cerebral vasohemodynamics (Table 1).

**Table 1:** Potential target areas, molecular pathways, cellular targets, and target molecules for understanding fatigue in individuals with autoimmune and related disorders.

**Potential target areas**	**Related molecular pathways**	**Cellular targets**	**Target molecules**
Inflammatory-related molecules	- AP-1 pathway - B-cell receptor signaling - COX-2/prostaglandins - NLRP3 inflammasome pathways - JAK/STAT pathway - MAPK pathway - NF-κB - T-cell receptor signaling - Vagal afferents - Vagal efferents	- Astrocytes - B-cells - Endothelial cells - Macrophages - Microglia - Neurons - Pericytes - Perivascular macrophages - T-cells	- IL-1β - IL-6 - IL-18 - IFN-γ - TNF-α - Other cytokines/chemokines
Metabolic-related molecules	- Brain glymphatic system - Citric acid cycle - Gluconeogenesis - Glycolysis - Oxidative phosphorylation - NLRP3 inflammasome pathways - Pentose phosphate pathway	- Astrocytes - Microglia - Neurons - Pericytes - Perivascular macrophages	-Acetyl-CoA - ATP - Hexokinase - NADH - Purinergic receptors
Sleep-related molecules	- ARAS - Neurotransmitter pathways - NLRP3 inflammasome pathways	- Astrocytes - Microglia - Neurons	- IL-1β - Neurotransmitters - TNF-α
Circadian-related molecules	- CLOCK pathway	- Astrocytes - Microglia - Neurons	-BMAL1 - CLOCK - PERIOD
Stress-related molecules	- HPA-axis pathway - Sympathomedullary pathway	- Astrocytes - Macrophages - Microglia - Neurons	-Adrenocorticotrophic hormone - Catecholamines - Corticosteroids - Corticotrophin - IL-1β - TNF-α
Vasoregulatory-related molecules	- Adrenergic cAMP and protein kinase A - cGMP - Protein kinase G - Neurotransmitter pathways - Voltage-sensitive calcium channels	- Astrocytes - Endothelial cells - Interneurons - Microglia - Neurons - Perivascular macrophages - Pericytes	- Adenosine - Dopamine - Epinephrine - IL-1β - Nitric oxide - Norepinephrine - TNF-α

## Inflammation

Autoimmune diseases are associated with enhanced pro-inflammatory signals in the periphery and CNS (33–37). The location of the enhanced inflammation varies with the type of autoimmune disease and the progression of the condition. Fatigue is observed in non-autoimmune disease and related conditions that have increased inflammation in the periphery and/or CNS, including cancer (38), sleep disorders (31, 39, 40), stroke (41, 42), and traumatic brain injury (43, 44). Several non-inflammatory factors are known to be affect fatigue including impairments in hydration status (45), pain (46), interactions from pharmaceuticals (47), muscle/exercise (48), hypothyroidism (49), radiation therapy (50), lung function, and cardiovascular characteristics such as blood pressure, heart rate, cardiac output, and stroke volume (51, 52). Many of the non-inflammatory components contributing to fatigue are modulated by or modulate inflammatory processes. Regardless, inflammatory mediators are reported to affect different aspects that contribute to fatigue including motivation (53), sleepiness (54, 55), cognition (56), anxiety (57), depression (58, 59), and stress (60).

Cytokines are small proteins molecules involved in cell signaling allowing cells to communicate through autocrine, paracrine, or endocrine mechanisms (61). Cytokines modulate immune responses, inflammation, cell growth and maturation, and normal physiological functions. They are highly conserved among species ranging from invertebrates to rodents and humans. Inflammatory cytokines are produced by nucleated cell types including lymphocytes and macrophages, as well as microglia, astrocytes, and neurons in the CNS (62). Inflammatory mechanisms involved in the etiology of fatigue implicate a significant involvement of cytokines. Interleukin (IL)-1 beta (IL-1β), tumor necrosis factor-alpha (TNF-α), IL-6, and interferon-gamma (IFN-γ) are cytokines that all have pro-inflammatory properties, and their enhancement are the most well-characterized in inducing fatigue and/or altering aspects contributing to fatigue (25, 63).

Autoimmune disease induces enhancement in cytokines such as IL-1β, TNF-α, IL-6, IL-12, IL-23, and IFN-γ, especially by T helper cells and macrophages (64, 65). Consequently, treatments targeting aspects of lymphocyte regulatory processes benefit the treatment of autoimmune diseases including Sjörgen's syndrome, rheumatoid arthritis, and inflammatory bowel disease. Macrophage activity is intertwined with T-cell functions (66), and macrophages are involved in the pathogenesis of autoimmune diseases (65). Macrophages in an M1 classical activated state tend to induce a pro-inflammatory response including IL-1β, TNF-α, IL-6, IFN-γ (67). Macrophages in a M2 alternatively activated state tend to have anti-inflammatory properties expressing IL-10, transforming growth factor-beta (TGF-β), and IL-1 receptor antagonist (IL-1RA). Pro- and anti-inflammatory cytokines regulate a balance in inflammatory status and the production of other cytokines. In addition, the overall level of localized cytokine secretion or its persistent enhancement or attenuation can lead to the upregulation or downregulation of associated receptors to modulate the downstream mechanisms or production of cytokines. Cytokines regulate normal physiological functions including mood, cognition, and sleep, and their expression varies over the course of the day and in response to local activity (30). Consequently, it is likely that dysregulation in inflammatory cytokines and their receptors in autoimmune disease serves to disrupt the normal physiological homeostasis of cytokines and contribute to fatigue.

Inflammation in the periphery can induce inflammation in the CNS and sickness behaviors (68, 69), which are behaviors associated with different aspects of fatigue (70). Experimental studies in rodents applying IL-1β, TNF-α, or IL-6 or substances that enhance their activity, such as the gram-negative bacterial cell wall component lipopolysaccharide (LPS), to the periphery or CNS result in alterations in behaviors affecting fatigue (30, 71, 72). Such applications can impair behaviors associated with fatigue including cognition, mood, pain, sleep, and motivation. Herein, we give several examples of how IL-1β modulates behaviors that can alter fatigue. Dysregulation of the IL-1 cytokine family member IL-1β or its receptor is implicated in fatigue in many autoimmune diseases (73). Enhanced IL-1β and its receptors are also implicated in fatigue (74). Nevertheless, evidence also suggests that IL-1β signaling is not necessary for other types of fatigue, including conditions like tumor-associated fatigue (75). Collectively, these findings from the animal literature indicate redundancy in how inflammatory cytokine affects fatigue-related behavior, although differences likely relate to the brain area and cells affected, amount of cytokine activity, timing of the cytokine activity, and interactions with normal physiological activities.

IL-1β binds to IL-1 receptor type I (IL-1R1) to induce inflammatory effects (76). IL-1β can also bind to IL-1 receptor type II, which acts as a decoy to inhibit IL-1β activity. Additionally, IL-1RA can inhibit IL-1β functioning by binding the IL-1R, thus preventing subsequent signaling processes. IL-1RI is functional in the presence of IL-1 receptor accessory protein (IL-1RAP). Activation of the IL-1RI promotes myeloid differentiation primary response 88 (MYD88), which subsequently activates the interleukin-1 receptor associated kinase 4 (IRAK4) (77). This activation ultimately leads to an enhancement of inhibitor of kappa B kinase (IKK) and mitogen-activated protein kinase (MAPK) kinase (MAPKK). IKK then can function to induce the inhibitory kappa B (IκB) to be released from nuclear factor kappa B (NF-κB), which allows NF-κB to translocate the nucleus and induce the transcription of pro-inflammatory molecules and processes. MAPKK induces c-Jun N-terminal nucleated kinase (JNK) and p38 to translocate the nucleus inducing activator protein-1 (AP-1) transcription of pro-inflammatory molecules and processes (78, 79). Interestingly, an IL-1 receptor accessor protein that is primarily expressed in the CNS (IL-1RAPb) was identified (80). IL-1RAPb binds to IL-1RI and inhibits the responses of the downstream adaptor molecules MYD88 and kinase IRAK4 (80, 81). Evidence suggests that the IL-1RAP is involved in modulating behavior under conditions of enhanced inflammation (82). Thus, although speculative, it is plausible that IL-1RAPb is involved in modulating cytokines involved in fatigue.

Cognitive fatigue involves declines in alertness, orientation, and mental performance on cognitive tasks and is associated with feelings of exhaustion that follow sustained cognitive demands (83). Individuals with autoimmune diseases, such as multiple sclerosis, often experience cognitive deficits and increased perceived cognitive fatigue associated with impaired cortical brain activity as determined using near-infrared spectroscopy (83). While there is a lack of specific tests in animal models regarding cognitive fatigue, the relationship between pro-inflammatory cytokines, especially IL-1β, and cognition is described (84, 85). Increased neuronal activity is observed with cognitive activities or whisker stimulation (86, 87), which enhance the expression of IL-1β or TNF-α in corresponding brain areas or barrel cortices (88, 89), respectively. In rodents, intracerebroventricular (ICV) or intraperitoneal (IP) injections of IL-1β prior to memory training impairs subsequent cognitive performance using the Morris water maze (90), a test of spatial memory that utilizes the hippocampus (91), or the eight-arm radial maze which tests working memory (92). The area of the brain where the inflammation occurs mediates the effect of the behavioral impairments. This is observed, for example, by the infusion of IL-1β locally into the hippocampus, which impairs working memory in a hippocampal-dependent memory task such as the three-panel runaway task administered to rats (93). Disease-specific aspects of autoimmune disease can also influence types of performance decrements. For instance, ICV injection of Human Immunodeficiency Virus-1 (HIV-1) envelope glycoprotein 120, a molecule that enhances IL-1β and TNF-α in individuals with HIV, increases IL-1β levels in the hippocampus and impairs contextual memory performance in rats (94). Inhibition of ligands for pro-inflammatory cytokines including IL-1β, TNF-α, IL-6, and IFN-γ using transgenic knockout mice, siRNA, or more cutting-edge technologies such as optogenetics or chemogenetics further implicate the role of inflammation in altering components related to fatigue (70, 95, 96). IL-1R1 knockout mice or an IL-1RA applied to the circulation given peripheral LPS demonstrate cognitive dysfunction associated with a fear conditioning test suggesting that reduced activation of peripheral inflammatory activity can inhibit central mediated behavior (97). However, evidence in animals also indicates that pro-inflammatory cytokines are required for normal behavioral functions and that an optimal zone exists for proper functioning (70, 84). This effect is seen in mice lacking IL-1R1 or mice given an IL-1RA to the periphery, which demonstrate reduced cognitive responses in the Morris water maze (90, 98, 99). Nevertheless, other studies demonstrate mice lacking IL-1R1 demonstrate normal learning in cognitive tests including the Y-maze, T-maze, and Morris water maze (100).

Autoimmune diseases including inflammatory bowel disease, multiple sclerosis, and rheumatoid arthritis have a high comorbidity with anxiety, depression, and pain (14), which can serve to induce fatigue. A study involving ICV injections of IL-1β demonstrated increased anxiety-like behavior in mice determined by shorter time spent in the center area of the open field test and increased time spent in the closed arms of the elevated plus maze (101). Additionally, central administration of IL-1β increases immobilization periods in the tail suspension test (102), which assesses depressive-like behavior in mice. Evidence also suggests that social aspects are impaired with enhanced CNS inflammation. For example, ICV injections of IL-1β reduces social interactions as evidenced by reductions in social exploration behavior including active investigation, anogenital sniffing, and wrestling in rats (103). Furthermore, mice lacking the IL-1R1 demonstrate anxiogenic-like behavior with increased time spent in the open arms of the elevated-plus maze (100). However, IL-1R1 knockout mice are reported to not exhibit reduced mobility in a forced swim test, which assesses depressive like behavior in rodents (100).

The perception of effort and motivation can modify fatigue and are affected in autoimmune disease. Animal studies indicate that effort expenditure is influenced by inflammation (104–106). In general, inflammation increases averseness toward negative stimuli and positive stimuli (107). Animal models of motivation indicate that both approach and avoidance motivation are affected by pro-inflammatory cytokines. For example, IP injections of IL-1β impairs motivation in rats as observed with reduced lever pressing in a chow feeding choice procedure (105). IL-1β applied centrally in rats that are food-deprived impairs rodent choice toward favoring a low-effort, low-reward option using a two-choice high vs. low effort/reward task (105). These findings suggest that IL-1β can influence motivation away from high effort/high award tasks, although in this study the use of operant responding for food pellets could be interpreted as IL-1β influencing consummatory behavior. However, in a study where mice received IP injections of LPS with free-feeding, the mice exhibited a reduction in nose pokes for the low-effort, low-reward stimuli of grain resulting in an increase in percentage of high-effort, high-reward stimuli for earned chocolate (108). These data suggest that inflammation affects incentive motivation, in part, though altering willingness to exert effort for reward instead of reducing sensitivity to the reward, although the differences in effects could also be due to species differences or additional cytokines and inflammatory pathways that LPS activates.

**Figure 1:**
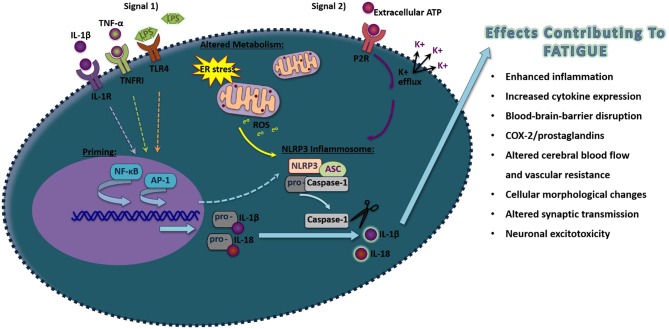
Schematic of proposed NLRP3 inflammasome activation in inducing pro-inflammatory molecules that induce fatigue. Exposure to pathogen-associated molecular pattern or danger-associated molecular pattern that act on their pattern recognition receptors (Signal 1), such as LPS, TNF-α, or IL-1β acting through Toll-like receptor 4, TNFR1, and IL-1R, respectively, will activate the transcriptional factors NF-κB that will prime components of the NLRP3 inflammasome. Additionally, transcription of inflammasome components can also occur through the activation of AP-1. A secondary signal is then required to activate inflammasome formation, including by alterations in metabolism that induce ROS or the activation of the purine type 2X receptors (P2XRs). Upon formation of NLRP3, ASC, and pro-caspase-1, caspase-1 will be allowed to disassociate and cleave the inactive pro-forms of IL-1β and IL-18 into their mature active forms. Upon release, these pro-inflammatory cytokines can alter surrounding cells leading to effects contributing to fatigue.

A primary mechanism that activates IL-1β occurs through the activation of inflammasomes (109). Inflammasomes are large intracellular signaling protein complexes found within the cytoplasm of most nucleated cells including neurons, astrocytes, microglia, and perivascular macrophages in the brain (Figure 1) (44, 109, 110). Inflammasome activation involves a priming step and a secondary step that induces the formation of the complex to activate caspase-1 to cleave the pro-forms of IL-1β and other IL-1 family members, such as IL-18 and IL-33, into their mature active forms (111). The priming signal involves the activation of transcriptional processes such as NF-κB or AP-1 to produce the components of the inflammasome as well as the pro-forms of the cytokines that will be activated upon inflammasome formation and subsequent caspase-1 release (112, 113). Inflammasome priming can occur by the activation of different types of receptors including the IL-1RI by IL-1β, TNF receptor I by TNF-α, or the toll-like receptor 4 (TLR4) by LPS to activate NF-κB (114). JNKs and MAPK/extracellular signal regulated kinase (ERK) pathways, which can activate AP-1 mediated transcription, also are implicated in the activation of inflammasomes (115). Most inflammasomes contain a nucleotide-binding oligomerization domain-like receptor or an absent in melanoma 2 (AIM2)-like receptor (109). The nucleotide leucine-rich protein-3 (NLRP3) inflammasome is the most widely characterized inflammasome, although multiple types of inflammasomes exist with unique recognition abilities in response to specific pathogen-associated molecular patterns (PAMPS) or danger-associated molecular patterns (DAMPS) (111). PAMPs and DAMPs include components of pathogens, energy-related molecules, double-stranded or single-stranded deoxyribonucleic acid (DNA), ribonucleic acid (RNA), or chemical substances. PAMPs and DAMPs are recognized by their specific associated pattern recognition receptor (PRR). These processes lead to the recruitment of the adapter apoptosis-associated speck-like protein containing a C-terminal caspase recruitment domain (ASC; also known as pycard1) and pro-caspase-1, which conglomerate to activate caspase-1.

Inflammasomes are involved in modulating behavior including sleep (116), cognition (117), anxiety (118–120), and depression (120). Evidence also suggests that inflammasomes are hyper-activated in individuals with autoimmune diseases such as rheumatoid arthritis, systemic lupus erythematosus, spondyloarthritis, Sjogren's syndrome, and Crohn's disease (121, 122). Fatigue-related tests in mice including the repeated forced swim test, locomotor activity, and Rota-rod test have shown impaired performance associated with enhanced activation of inflammasome-related molecules, and mice lacking NLRP3 have demonstrated reduced fatigue-like behavior (123). TNF-α and IFN-γ have unique inflammatory pathways involved in their activation and are indirectly involved in mechanisms that can induce inflammasome activation (124, 125). Evidence also suggests that IL-1β can enhance reactive oxygen species (ROS), in part, through increasing the phosphatidylinositol 3-kinase (PI3-K)/protein kinase B (AKT)/mammalian target of the rapamycin (mTOR) pathway—a pathway implicated in inflammasome activation (125–127). Hexokinase-1 is also implicated in mTOR and NLRP3 inflammasome activation (128, 129).

IFN-γ is involved in innate and adaptive immunity. IFN-γ and its ligands (the IFN-γ receptor 1 and IFN-γ receptor 2) are found throughout tissues in the periphery and CNS, especially in macrophages, microglia, and lymphocytes (130). IFN-γ receptors stimulate the Janus kinase (JAK)-signal transducer and activator of transcription protein (STAT) pathway (130). The JAK-STAT pathway can interconnect with other inflammatory pathways including the PI3K/AKT/mTOR pathway and the MAPK/ERK pathway, which can serve to ultimately enhance NLRP3 inflammasome activation (130). Additional evidence suggests that IFN-γ effects on IL-1β are related to the enhancement of suppressor of cytokine signaling 1 (SOCS1) (131). Direct evidence also indicates that IFN-γ upregulates NLRP3, ASC and pro-caspase 1 expression (125).

## Metabolism

Metabolism involves the conversion of fuel sources to energy-related molecules—such as adenosine triphosphate (ATP) and nicotinamide adenine dinucleotide phosphate (NADPH)—enabling cellular processes; synthesis of components for proteins, lipids, nucleic acids, and carbohydrates; and the removal of ROS such as peroxides, superoxide, and hydroxyl radicals (132). Metabolism is involved in mechanisms that enhance pro-inflammatory cytokines including IL-1β, TNF-α, IL-6, and IFN-γ (133, 134). Alterations in metabolism have been implicated in sleep regulation and fatigue (135, 136). Metabolism also is tied to glycogen metabolism, glycogen synthesis, and energy substrates and their derivatives (132, 135), which could potentially alter fatigue. Alterations in metabolism are also implicated in the pathogenesis of autoimmune disease (63).

ATP is an energy molecule involved in the catabolism of macronutrients including carbohydrates, proteins, and lipids (137). ATP is modulated by intracellular glucose availability and mitochondria production (137). ATP can function as a neurotransmitter, in part, by acting on purine type 2 receptors (138, 139). Also, ATP can be co-released in to the extracellular space with other neurotransmitters such as norepinephrine or acetylcholine (138, 139). A major mechanism in the activation of NLRP3 inflammasomes involves extracellular ATP acting through purine type 2 (P2) receptors, including the P2X7 receptor, to induce potassium efflux leading to the formation of the NLRP3 inflammasome (140). Nevertheless, ATP is rapidly metabolized to adenosine diphosphate and adenosine monophosphate by ectonucleoside diphosphohydrolase (i.e., CD39). AMP is then metabolized to adenosine by ecto-5′ nucleotidase (i.e., CD73) (141), which is a molecule involved in modulating sleep (142). Adenosine acts on purine type 1 receptors, is involved in sleep regulation, and inhibits arousal (143), which could potentially lead to fatigue. Inhibition of purine type 1 receptors with adenosine 2A receptor antagonists or caffeine can enhance wakefulness and reduce some types of fatigue (144), although the persistent use of caffeine can be detrimental to sleep (145).

ROS are involved in cell signaling, homeostasis, and normal physiological processes including inflammatory processes (146). Evidence in animal models and patients indicates that increased ROS activity is involved in the pathogenesis of autoimmune diseases (147). ROS are formed from the metabolism of oxygen during cellular respiration (148). ROS production—done with the aid of enzymes including cyclooxygenase, lipoxygenase, NAPDH oxidases, and xanthine oxidase—can lead to enhanced pro-inflammatory cytokine production (149). Within the CNS, neurons largely rely on oxidative phosphorylation (OXPHOS) for ATP requirements (150). Neurons work synergistically with astrocytes—cells that utilize glycolysis to convert lipids and glucose to pyruvate, which is ultimately converted to lactate (151). Glycolysis and oxidative phosphorylation generate two ATP molecules per glucose molecule and 36 ATP molecules for OXPHOS, respectively (152). However, glycolysis also produces substrates for pentose phosphate pathway (PPP) to make ribose 5-phosphate for the generation of NADPH (153). NADPH oxidase aids in the production of ROS in microglia, astrocytes and neurons (110). Major areas of activation in the mitochondrial respiratory chain are at complex I and complex III. Electrons from NADH are accepted in complex I where they move through an electrochemical gradient to complex II through ubiquinone (154). Thereafter, the electrons are moved to cytochrome C and complex IV where oxygen is converted into water, which can lead to pro-inflammatory cytokine production (155, 156).

Evidence indicates that NADPH oxidases are activators of NLRP3 inflammasomes (157). In particular, cells undergoing ROS that are primed for NLRP3 activation enhance redox-dependent transcription factors including NF-κB (158). Other studies indicate that IL-1β and caspase-1 are ROS-dependent (159). IL-1β also enhances intracellular ROS by uncoupling superoxide dismutase, catalase, and glutathione peroxidase, which have anti-oxidant functions, thus inducing a feedforward production in inflammasome activation (115). ROS inhibitors also can reduce the secretion of IL-1β. NADPH oxidase appears to be a primary contributor for the ROS activation of inflammasomes involving extracellular ATP, although other contributors are also likely. This is consistent with studies indicating that the enhancement of ROS is dependent on P2X7 receptor activation (160). Moreover, the NADPH inhibitor diphenyleneiodonium attenuates caspase-1 activation by ATP further suggesting energy- and ROS-related mechanisms in inflammasome activation (115).

Mitochondrial DNA dysregulation has also been observed in inflammatory conditions including autoimmune diseases such as systemic lupus erythematosus, rheumatoid arthritis, and granulomatosis with polyangiitis (161). Mitochondrial stress can induce the release of mitochondrial DNA into the cytosol, resulting in increased NLRP3 inflammasome activation (162). Mitochondrial DNA can be altered by the transcription factor A mitochondrial to impair OXPHOS (163). In addition, evidence indicates that the escape of mitochondrial DNA into the cytosol involves the DNA sensor cyclic guanosine monophosphate-adenosine monophosphate synthase (cGAS), which promotes stimulator of interferon genes (STING)-interferon regulatory factor 3 (IRF3) dependent signaling to increase the expression of interferon-stimulated genes (164). STINGs are cytosolic proteins that are attached to the endoplasmic reticulum. STING activates IRF3 which then translocates to the nucleus to transcribe type I IFN genes and NF-κB. Interestingly, recent evidence suggests that IRF3 in antigen presenting cells and T-cells that is necessary for maximized IFN-γ responses (165).

Thioredoxin (TRX) and its endogenous inhibitor thioredoxin-interactive protein (TXNIP) are involved in the activation of inflammasomes (111). TXNIP, when released from the oxidized TRX, will bind directly to leucine-rich areas of NLRP3 inducing the inflammasomes to form (111). Interestingly, multiple sclerosis patients that were not exposed to interferon and immunosuppressive treatments exhibited significant enhancements in TRX expression in peripheral blood mononuclear cells compared to healthy controls while multiple sclerosis patients that did undergo treatments did not show significant differences (166). Moreover, all multiple sclerosis patients regardless of treatment exhibited greater TXNIP expression values (166).

The mechanistic target of rapamycin (mTORC) is a major sensor of metabolic stress and the pro-inflammatory response (167, 168). Evidence indicates that the mTORC1 complex is activated by mitochondrial ROS. Interestingly, mTORC1 and mitochondrial ROS are known to activate NLRP3 inflammasomes in a mouse model of lupus (127). The mTORC1 complex is also coupled with the metabolic depletion of glutathione (169). The depletion of glutathione can be reversed with N-acetylcysteine (NAC) by reducing cysteine and NADPH and inhibiting mTORC1 (169). NAC has been shown to be beneficial in blocking mTOR in systemic lupus erythematosus patients (170).

Cyclooxygenase (COX) converts prostaglandins from arachidonic acid (171). COX-2 is inducible and involved in modulating inflammatory mediators including IL-1β, while COX-1 is constitutively expressed (171). COX-2 is found in most cells including neurons, perivascular cells, and endothelial cells (171). The initiation and resolution of inflammation and alteration of autoimmune-related immunity is modulated by prostaglandin E2, in part, by IL-22 production, T helper 1 cell differentiation and Th17 cell proliferation (172). Evidence suggests that prostaglandin E2 is involved with inflammatory stimuli-induced fatigue (172). These findings collectively suggest that COX-prostaglandin E2 could be involved in fatigue occurring with autoimmune disease. Nevertheless, COX-2 inhibitors tend to have modest effects on fatigue, which could be related to its local cellular effects.

## Sleep and Circadian Rhythms

A bidirectional relationship appears to exist between sleep and circadian disturbances with autoimmune disease (173–175). Chronic insomnia is associated with an increased incidence of developing autoimmune disease (176). Findings in animal model of systemic lupus erythematosus also suggest that sleep deprivation could be involved in the etiology of the disease (177). Short sleep duration of <7 h of sleep per night is also associated with transitioning to systemic lupus erythematosus (178). Evidence also suggests that the sleep disorder narcolepsy has an autoimmune origin (179). Individuals with autoimmune disease often report disturbed sleep (180–185). In a mouse model of systemic lupus erythematosus, it was reported that an increased disturbances in sleep corresponded with the progression of the disease including increased sleep fragmentation and impaired sleep-stage transitions (186). The comorbidity of autoimmune disease and sleep disorders is documented for a small number of autoimmune diseases, although this area of research is widely under investigated. Rheumatoid arthritis, ankylosing spondylitis, systemic lupus erythematosus, Sjörgen's syndrome, pemphigus, and systemic sclerosis are associated with increased occurrence of sleep apnea (174, 187, 188). Multiple sclerosis is associated with an increased risk of rapid-eye movement (REM) sleep behavior disorder (189). Increased risk of restless legs syndrome is associated with autoimmune disorders including multiple sclerosis, psoriasis, and rheumatoid arthritis (190–192). Clinical diagnosis of chronic fatigue syndrome/myalgic encephalomyelitis is also partially based upon non-restorative sleep suggesting relationships between poor sleep and fatigue (193). In addition, secondary effects of autoimmune disease or sleep disorder pathology might serve to affect sleep. For example, chronic pain is found in individuals with autoimmune disease (194). Up to 88% of individuals with chronic pain also report disturbed sleep (195). Conversely, it is reported that up to 50% of individuals with insomnia also indicate enhanced pain (195), which could contribute to fatigue.

Sleepiness, often referred to as drowsiness, can be loosely defined as the inability to remain awake or the enhanced occurrence or compulsion to sleep. Sleep disorders and disturbed sleep are associated with sleepiness (196). Sleepiness can impact vigilance, cognition, mood, and attention as well as induce fatigue (197). Many sleep-related pathologies, including insomnia and sleep apnea, are associated with impairments in vigilance, cognition, mood, motivation, and attention (198–201). Sleepiness can be induced by acutely staying awake for extended periods of time or by the fragmented sleep that occurs chronically with many health conditions including sleep apnea and autoimmune diseases (202, 203). Evidence suggests that there is a dose-dependent impact whereby greater amounts of sleep loss correlate with enhanced sleepiness and poor performance (204). Nevertheless, sleepiness varies over the time of day (205), which can impair an individual's ability to sleep or the effectiveness of naps in preventing detriments of sleepiness in functional tasks such as cognition (206). Individuals experiencing fatigue often experience waves of periods of times when their fatigue is worse (207). Furthermore, individuals with multiple sclerosis may experience increased incidence of fatigue in the morning while others do so in the evening (207). Although speculative, prior sleep or circadian factors are likely contributors to these time-of-day fluctuations.

Sleep disorders—such as sleep apnea, insomnia, and REM sleep behavior disorder—are associated with enhanced levels of inflammatory molecules (208–210). Since inflammation can induce fatigue, it is plausible that neuroinflammation occurring with disturbed sleep or sleep loss could exacerbate fatigue in individuals with autoimmune disease. In a variety of species—including rats, mice, and rabbits—pro-inflammatory cytokines can enhance non-rapid-eye movement sleep when applied to the periphery or CNS (30, 211), although IL-1β and TNF-α are the most thoroughly investigated. When IL-1β or TNF-α are applied to the CNS they also enhance electroencephalogram delta activity (~0.5–4 Hz frequency range) (30, 211), a frequency of brain electrical activity that is associated with an increased pressure to sleep after sleep loss. Additionally, conditions that induce sleepiness—including sleep loss, pathogens, and related components such as influenza and LPS—enhance IL-1β and TNF-α in the brain (30, 211). However, inflammatory cytokines, such as IL-1β do not remain in a steady state throughout the course of a day (211). Inhibition of pro-inflammatory cytokines or their receptors via pharmaceuticals, siRNA, or knockout animals can inhibit enhanced sleepiness by pro-somnogenic substances (30, 211). Collectively, these findings supporting the idea that dysregulated homeostatic cytokine expression in autoimmune disease contributes, in part, to sleep disturbances in individuals with autoimmune disorders. Moreover, it is reasonable that sleep loss, which enhances pro-inflammatory cytokines, could exacerbate sleep disturbances in autoimmune disease. Indeed, prolonged sleep deprivation using a multiple platform method in an animal model of systemic lupus erythematosus using New Zealand Black/New Zealand White F1 mice were shown to exhibit earlier onset of disease like symptoms (177).

Recently, the NLRP3 inflammasome was found to be critical to sleep and electroencephalogram delta power fluctuations throughout the day as well as responses to sleep loss and inflammatory stimuli in mice (212). Additionally, infusing a caspase-1 inhibitor ICV into rats attenuated sleep and electroencephalogram delta power responses to LPS (213), further suggesting the involvement of the NLRP3 inflammasome in sleep regulation. Moreover, evidence indicates that there is an upregulation of NLRP3 inflammasome related components with sleep deprivation and sleep fragmentation (214–216). In the cortex, there are diurnal variations in NLRP3 expression, IL-1β protein, and caspase-1 activity occurring at times of day following high neuronal activity when sleep propensity is the greatest indicating a relationship between activity and inflammation in the CNS (212).

The transcriptional factors brain and muscle aryl hydrocarbon receptor nuclear translocator-like protein 1 (BMAL1) and CLOCK (CLK) form heterodimers in the cytoplasm that regulate gene expression oscillator properties of cells that regulate both the persistence and duration of circadian rhythms (217). BMAL1 and CLK recruit the co-activator cyclic AMP response element-binding protein (CREB)-binding protein and, upon phosphorylation, form a CLK/CYCLE (CYC) complex that binds to the E-box of the promoter PERIOD (PER) (218), which enhances the expression of these promoters and modulates the circadian system. The CLK gene is a major transcription factor that serves as a circadian pacemaker and is a co-activator of CREB and modulates PER protein (219). CLK also induces histone acetyl transferase activity (220), which enhances the dimerization of BMAL1 and is associated with binding to another cytokine implicated in fatigue, IL-1alpha (221, 222). Clock gene expression is enhanced in a dosage-dependent manor with increased oxygen concentrations (223). Furthermore, mice lacking NLRP3 can inhibit CLK gene in hyperoxia-induced lung inflammation (223). An animal model of autoimmune encephalomyelitis found that within the CNS, the enhanced infiltration of IL-1β secreting CD11b/Ly6Chi monocytes enhanced IL17^+^/IFN-γ^+^ T cells with the loss of myeloid BMAL1 at midday instead of midnight (224). These data suggest that IL-1β and inflammasome activation or dysregulation could alter T cell cytokine responses to further dysregulate sleep and/or fatigue.

Melatonin is secreted by the pineal gland, regulates circadian rhythms, and has anti-inflammatory and anti-oxidant properties (225). Melatonin receptors are expressed on CD4 and CD8 T-cells and B-cells. Additional evidence suggests that melatonin attenuates the expression of IL-1β, TNF-α, IL-6, and IFN-γ (225). The anti-inflammatory properties of melatonin occur, in part, through the inhibition of NF-κB (225). An association between multiple sclerosis and melatonin has been observed clinically (225). Melatonin therapy has been shown to attenuate inflammatory cytokines and related pathways in both animal models and human studies of multiple sclerosis, type 1 diabetes, inflammatory bowel disease, and systemic lupus erythematosus (225), and thus could potentially be beneficial in combating fatigue.

## Stress

The hypothalamic-pituitary-adrenal (HPA) axis involves interactions between the hypothalamus and pituitary glands and is involved in hormonal responses to stress that modulate fatigue (226, 227). Individuals with autoimmune disease are reported to have increased stress levels compared to the general population. Evidence indicates that stress can modulate brain inflammation in autoimmune diseases such as multiple scleorsis (228). Sympathomedullary system activation will release norepinephrine (also referred to as noradrenaline) from nerve terminals and epinephrine (also called adrenaline) from the adrenal medulla (229). These catecholamines can increase heart rate and blood pressure.

Microglia, macrophages, and astrocytes in the central nervous system express adrenaline receptors (230). Adrenaline receptors are altered by complement-induced innate immune responses. Interestingly, several autoimmune and related disorders are associated with dysregulated complement including Sjögren's syndrome, rheumatoid arthritis, and systemic lupus erythematosus (231). Evidence indicates that activated microglia are selectively inhibited by norepinephrine (232). Furthermore, norepinephrine applied with ATP attenuates the baseline rate and increased ATP-induced process extension and migration of microglia *in vitro* suggesting relationships exist between the activation of purinergic and adrenergic systems (233). Beta-adrenergic receptors also exhibit an anti-inflammatory influence on astrocytes by reducing TNF-α-related genes including IL-6, CXCL2, CXCL3, VCAM1, and ICAM1 expression (234).

Within the hypothalamus, stress induces the release of corticotrophin-releasing hormone (CRH) (229). CRH induces the release of adrenocorticotropic hormone (ACTH) from the pituitary, which enhances the release of glucocorticoids from the adrenal cortex (229). Glucocorticoids have an anti-inflammatory function that occurs, in part, by inhibiting IL-1β, IL-6, and IFN-γ, as well as cyclooxygenases and prostaglandins (229, 235). However, a relationship exists between cytokines and ACTH and glucocorticoids whereby IL-1β, IL-6 and TNF-α can stimulate the pituitary adrenal axis to increase serum levels of ACTH and glucocorticoids (229, 235), which will inhibit pro-inflammatory molecules. Nevertheless, glucocorticoids can also have pro-inflammatory influence on the immune system (235). Glucocorticoids can enhance the expression of NLRP3 to enhance IL-1β responses to ATP (236). Glucocorticoids are highly expressed in the hippocampus and prefrontal cortex and thus could modulate the inflammatory response for behaviors such as cognitive fatigue (229). Stress-related molecules including glucocorticoids exhibit diurnal patterns during which they tend to be greatest during the inactive period of the day (237). Sleep loss also can enhances levels of the glucocorticoid cortisol (238). Thus, the effects of stress, sleep, and fatigue are intertwining. Chronic stress, such as those undergoing chronic health conditions, can reduce glucocorticoid sensitivity to promote inflammatory signaling (239). This can occur, in part, the suppression of cortisol signaling sensitivity, the functional heterogeneity of monocytes, or the diminished ability of monocytes to transduce cortisol signals—all of which could serve to enhance inflammatory cytokines (239).

The weak androgen dehydroepiandrosterone (DHEA) produced in the adrenal cortex. The HPA-axis, in part, controls DHEA synthesis (240). The greatest levels of DHEA demonstrate a circadian pattern similar to the pattern of ACTH secretion (240). DHEA can act as a positive allosteric modulator for the NMDA receptor and a negative allosteric modulator of the GABA_A_ receptor (240). DHEA is found to have reduced levels in autoimmune disease including multiple sclerosis, systemic lupus erythematosus, rheumatoid arthritis, and inflammatory bowel disease (241, 242). DHEA can function to attenuate the production of pro-inflammatory cytokines such as IL-1β and TNF-α though NF-κB (242). Collectively, stress can contribute to modulating brain inflammatory mediators, which could lead to exacerbation of molecules that can induce fatigue.

## Brain Areas and Neurotransmitters

Several brain areas are associated with modulating fatigue (Table 2) (243), although this area of research remains poorly investigated. These brain areas function through neurons and glia to release neurotransmitters (243). Neurotransmitters, especially monoamines, are potent at producing arousal, wakefulness, and motivation to perform activities when desired (30, 144, 244). Dopamine, norepinephrine, epinephrine, glutamate, and histamine are excitatory transmitters. Serotonin and gamma-aminobutyric acid (GABA) are inhibitory neurotransmitters that prevent neuron signals from continuing (144). There are many redundancies in the actions of these neurotransmitters, although there are also unique characteristics for receptors on the cells and brain areas that neurotransmitters act upon, as well as functional differences that exist based on the prior state of the brain cells and the concentration of the neurotransmitters. Inflammatory molecules are affected by neurotransmitters and vice versa (245), which can lead to behavior changes including those affecting fatigue.

**Table 2:** Overview of neurotransmitters, their receptors, brain areas, and associated fatigue-related behaviors.

**Neurotransmitter**	**Primarily excitatory or inhibitory**	**Receptors**	**Brain areas**	**Fatigue-related behaviors**
GABA	- Inhibitory	- GABA_A_ - GABA_B_	- Dorsal raphe nucleus - Lateral hypothalamus - Locus coeruleus - Periaqueductal gray area	- Arousal - Motivation - Sleep/wake -Sleepiness
Serotonin	- Inhibitory	- 5-HT receptors	- Dorsal raphe nucleus	- Arousal - Anxiety - Depression -Sleep/wake
Norepinephrine	- Excitatory	- Alpha-adrenergic receptors - Beta-adrenergic receptors	- Caudal ventral medulla - Nucleus tractus solitarius	- Anxiety - Attention - Depression - Motivation -Wakefulness
Dopamine	- Excitatory	- D1-like receptors - D2-like receptors	- Arcuate nucleus - Periventricular nucleus - Posterior hypothalamus - Subtantia nigra - Ventral tegmental area - Zona incerta	- Anxiety - Arousal - Depression - Motivation -Sleep/wake
Histamine	- Excitatory	- Histamine 1 receptors - Histamine 2 receptors - Histamine 3 receptors - Histamine 4 receptors	- Tuberomammillary nucleus	- Arousal - Mood - Sleep/wake
Glutamate	- Excitatory	- AMPA receptors - Kainite Receptors NMDA receptors	- Cerebellum - Cerebral cortex - Hippocampus - Parabrachial nucleus	- Attention - Cognition - Depression - Sleep/wake - Tiredness
Acetylcholine	- Excitatory	- Muscarinic acetylcholine receptors - Nicotinic acetylcholine receptors	- Basal forebrain - Cerebral cortex - Laterodorsal tegmental nuclei - Pedunculopontine nucleus	- Arousal - Cognition - Memory - Sleep/wake
Orexin	- Excitatory	- Orexin 1 receptors - Orexin 2 receptors	- Lateral hypothalamus - Perifornical area	- Arousal - Energy homeostasis - Sleep/wake

GABA is the major inhibitory neurotransmitter in the CNS (246). GABA ligands are GABA_A_ receptors that are ligand-gated ion channels and GABA_B_, which are metabotropic receptors that are G protein-coupled receptors that open and close ion channels. GABA functions to help neurons recover after transmission. It has been shown to reduce anxiety and stress and is involved in sleep and sleepiness (246, 247). The dorsal raphe nucleus, periaqueductal gray area, locus coeruleus, and lateral hypothalamus have high concentrations of GABAergic neurons, which are activated during NREM sleep (30). GABAergic neurons in the ventrolateral preoptic nucleus area of the lateral hypothalamus are involved in modulating NREM sleep. The ventrolateral preoptic nucleus also projects to areas of the hypothalamus, including the median preoptic nucleus, which is involved in NREM sleep (248). Ventrolateral preoptic nucleus neurons are inhibited by the neurotransmitters acetylcholine, norepinephrine, serotonin, and dopamine. Moreover, the ventrolateral preoptic nucleus projects to the locus coeruleus, periaqueductal gray matter, parabrachial nucleus, histaminergic cells in the tuberomammillary nucleus, and the dorsal raphe nucleus where it can function to inhibit those arousal-producing brain areas. Evidence also indicates that IL-1β and IL-1RA, respectively, enhance and attenuate GABAergic neuron action potentials (249), which, in part, could contribute to the attenuation of arousal producing neurons resulting in fatigue and sleepiness.

Serotonin is involved with altering anxiety, depression, sleep, and fatigue (250–252). A main area of serotonin production in the CNS is the raphe nuclei, located in the brainstem (253). Serotonin acts on 5-hydroxytryptamine (5-HT) receptors located on cell membrane of nerve cells. These receptors have unique and repetitive functions including altering dopamine release into the mesocorticolimbic pathway, acetylcholine release in the prefrontal cortex, activation of G_S_ signaling via activating adenylyl cyclase, and induction of vasoconstriction (254, 255). IL-1β is also associated with the release of serotonin, dopamine, and norepinephrine (256).

Norepinephrine is an excitatory neurotransmitter that binds to alpha- and beta-adrenergic receptors, which function as G protein-coupled receptors that act on secondary messenger systems (257). Norepinephrine is involved in anxiety and depression. However, low levels of norepinephrine are associated with fatigue and impaired motivation (258, 259). Neurons expressing norepinephrine are found in relatively small brain areas. The norepinephrine cell group A1 is located in the caudal ventrolateral part of the medulla within the brainstem (260). The norepinephrine cell group A2 is located in the brainstem area called the nucleus tractus solitarius (NTS) (260). Additionally, a major source of norepinephrine in the brain is the locus coeruleus, which is located in the pons of the brainstem (261). This area projects to all major parts of the brain including the cortex (262). Locus coeruleus activity is low during non-rapid-eye movement (NREM) sleep, with very little activity during REM sleep (262). Norepinephrine levels remain fairly constant during wakefulness but are enhanced when a stimulus is needed for reasons such as attention (262).

Dopamine is an excitatory neurotransmitter that binds to D1-like and D2-like receptors that increase or decrease intracellular amounts of cAMP by acting on adenylate cyclase and the trace amine-associated receptor 1 (263, 264). This increases intracellular amounts of cAMP and intracellular calcium levels (263). Dopamine is involved in motivation, interest, and drive (265). Low levels are found in individuals that experience difficulty completing tasks, poor concentration, no energy, and lack of motivation (266). Consequently, the dopamine imbalance hypothesis of fatigue suggests a U-shaped relationship between amounts of dopamine and levels of fatigue in which inadequate or excessive amounts of dopamine leads to fatigue (267). Main brain areas where dopaminergic neurons are found in the brain include the substantia nigra area of the basal ganglia, the ventral tegmental area (located near the midline of the midbrain of the brainstem), the posterior hypothalamus, the periventricular nucleus (located in the wall of the third ventricle within the hypothalamus), the arcuate nucleus area of the hypothalamus, the zona incerta (located below the thalamus), and the periventricular nucleus (268). The nigra-striatal pathway is heavily involved in motor functions. The ventral tegmental area dopaminergic neurons project to the prefrontal cortex in the mesocorticolimbic pathway while also projecting to the nucleus accumbens by the mesocorticolimbic pathway (268). The ventral tegmental area projects to the amygdala, cingulate gyrus (which is part of the cingulate cortex in the medial aspect of the cerebral cortex), hippocampus, and olfactory bulb (269, 270). The caudate is associated with cognitive fatigue in individuals with traumatic brain injury—a condition where inflammatory cytokines including TNF-α and IL-1β are enhanced (267, 271). Research suggests that individuals with multiple sclerosis have impaired dopamine function in the caudate (272).

Histamine is an excitatory neurotransmitter that acts on four G protein-coupled receptors (histamine 1, histamine 2, histamine 3, and histamine 4 receptors) (273). Histamine receptors function, in part, to activate phospholipase C, inhibit cAMP synthesis, and activate MAP kinases and the AKT/GSK3β pathway (274). Histamine can also affect sleep/wake, emotions, behavior, and fatigue (275, 276). Histamine is known to activate microglia leading to the production of pro-inflammatory cytokines including IL-6 and TNF-α (277). Histamine is also noted to be activated in chronic inflammatory diseases including autoimmune diseases such as multiple sclerosis (274). Histamine is involved in vasodilation and can alter blood pressure (273). Histamine also can function to release nitric oxide, which is a potent vasodilator (278). The tuberomammillary nucleus is the only known neuronal area to produce histamine (279). Histaminergic neurons project throughout the brain including the cortex and brainstem (280). However, histaminergic receptors are found on cells in other brain areas. Histamine 1 receptor is found on neurons in the tuberomammillary nucleus of the hypothalamus, which project to the dorsal raphe nucleus and locus coeruleus. The histamine 2 receptor is found in neurons in the dorsal striatum (caudate nucleus and putamen), external layers of the cerebral cortex, hippocampus, and dentate nucleus of the cerebellum. Histaminergic neurons within the tuberomammillary nucleus is involved in promoting arousal when activated (281). These neurons fire rapidly during wakefulness and stop during sleep (279). Histamine 1 receptor targeting drugs induce drowsiness, and over-the-counter anti-histamines are associated with increased sleepiness (282). Findings in experimental autoimmune encephalomyelitis (EAE), an animal model of enhanced brain inflammation, suggests that histamine acting through its histamine 1 and histamine 2 receptors can attenuate damage to the brain and inflammatory cytokines including IFN-γ (283, 284).

Glutamate is a major excitatory neurotransmitter found throughout the CNS that is produced by metabolism (285). Glutamate has affinity to bind to ionotropic and voltage-gated N-methyl-D-aspartate (NMDA) receptors that increase calcium membrane permeability; metabotropic glutamate receptors, which are G-coupled protein receptors that can activate phospholipase C or reduce intracellular levels of cAMP through adenylate cyclase; ionotropic alpha-amino-3-hydroxy-5-methyl-4-isoxazolepropionic acid (AMPA) receptors; and kainate receptors that increase sodium and potassium membrane permeability (286). Low levels of glutamate can lead to tiredness and poor brain activity (287). Glutamatergic neurons located in the parabrachial nucleus located in the dorsolateral pons of the brainstem and the adjacent precoeruleus area induce arousal through their projections to areas of the ascending reticular activating system (ARAS) (30). Glutamatergic neurons are also found in synapses within additional brain areas including the hippocampus, cerebellum and cerebral cortex. Extracellular glutamate concentrations operate within a tight physiological range involving its release and uptake to protect neurons from excitotoxicity (288). Dysregulation of glutamate can lead to diminishing the sodium-dependent glial glutamate transporter and impairing the activity of volume sensitive anion channels and hemi-channels of astrocyte to alter astrocytic end-feet (289). Evidence suggests that mGluR5 receptors mediate NF-κB, which is suggested to alter chronic inflammation associated fatigue (290). Ketamine is a potent NMDA receptor antagonist, which recent evidence indicates has anti-fatigue effects (291). Studies indicate that IL-1β can modulate the glutamate receptor NMDA and vice versa. The IL-1R1 is co-localized with the NMDA receptor NR2B subunit (292). IL-1β is enhanced in microglia and astrocytes within the cortex after NMDA induced excitotoxicity. Furthermore, inhibiting NMDA receptors in rats attenuates IL-1β expression after cerebral ischemia and high potassium cortical spreading and depression (293). Collectively, these studies suggest that altered inflammatory response, neuronal activity, and cerebral blood flow to modulate glutamatergic neuronal activity and possibly fatigue.

Acetylcholine is an excitatory neurotransmitter that is synthesized by choline acetyltransferase from choline and acetyl-CoA (294). Acetylcholinesterase transforms acetylcholine into acetate and choline. Acetylcholine binds to nicotinic acetylcholine receptors and muscarinic acetylcholine receptors (mAChR). Nicotinic acetylcholine receptors are ligand-gated ion channels that modulate the state of neurons by the movement of cations to induce depolarization of the plasma membrane. This results in an excitatory postsynaptic potential or activation of voltage-gated ion channels or leads to the entry of calcium and to the activation of intracellular cascades that can alter gene expression or the release of neurotransmitters. mAChR exhibit longer lasting actions and function as G protein-coupled receptors to induce their effects by second messenger systems. Acetylcholine is involved in both CNS and peripheral function including the neuromuscular junction. Acetylcholine regulates sleep/wakefulness, arousal, cognition, memory, and fatigue (295–297). Myasthenia gravis disease is an autoimmune disease in which nicotinic acetylcholine receptors are affected and a condition that is associated with fatigue and feelings of weakness (298, 299). Acetylcholinesterase inhibitors have been used to treat symptoms in conditions including Alzheimer's disease, Parkinson's disease, and schizophrenia (300). Cholinergic neurons in the brainstem including the pedunculopontine and laterodorsal tegmental nuclei as well as the basal forebrain are involved with arousal and wakefulness (296). These brain areas project to the lateral hypothalamus, prefrontal cortex, and reticular thalamic nuclei. Acetylcholine also acts on cells within the basal ganglia, locus coeruleus, dorsal raphe nucleus, and hippocampus. Inflammatory molecules can inhibit acetylcholine and vice versa. For example, acetylcholine significantly attenuates LPS-induced TNF-α production in microglia in a dosage dependent manor (301). In human and rat monocytes, acetylcholine also is effective in suppressing other endotoxin-inducible pro-inflammatory cytokines—such as IL-1β, IL-6, and IL-18—by a post-transcriptional mechanism (302). IL-1β can also inhibit acetylcholine synthesis in the brains stem (303).

Arousal and wakefulness involve an ascending pathway that begins in brainstem monoaminergic and cholinergic neurons located between the pons and the midbrain in the mesopontine junction (30). Fatigue is associated with brain areas that regulate arousal, attention, and reaction time. These areas include the ARAS, limbic system, anterior cingulate, and basal ganglia (304). Increased self-reported fatigue is associated with poor sleep quality or sleep disturbances (305), and this type of fatigue likely involves the ARAS system that modulates arousal and wakefulness. Cholinergic neurons in the pedunculopontine and laterodorsal tegmental nuclei of the brainstem are active during wakefulness, and their output traverses the mesopontine junction to the thalamus, as well as project to the lateral hypothalamus, basal forebrain, prefrontal cortex, and reticular thalamic nuclei (144). Neurons in the brainstem project to the basal forebrain and the lateral hypothalamus affecting arousal. The basal forebrain is in particular a brain area involved in mediating arousal. Diverse populations of neurons including GABAergic, glutamatergic, and cholinergic neurons serve to activate cortical pyramidal neurons to increase cortical activation as evidenced by electroencephalogram desynchronization. Cortical GABAergic interneurons—cells that are often activated during sleep—are inhibited by neurons of the basal forebrain.

Orexin (also known as hypocretin) is a neuropeptide that binds to orexin 1 and orexin 2 receptors (306). These neurons release acetylcholine, serotonin, and norepinephrine and are sensitive to metabotropic glutamate receptors, adenosine A1 receptors, muscarinic M3 receptors, serotonin 5-HT1A receptors, neuropeptide Y receptors, cholecystokinin A receptors, and catecholamines, as well as ghrelin, leptin and glucose (307). Since orexin produces monoamines that all involved with arousal and wakefulness and links exist between energy homeostasis and orexins (144, 308), it is plausible that orexinergic cells could be involved in aspects of fatigue. Orexin neurons are located in the perifornical area and the lateral hypothalamus (307). These neurons project throughout the cortex, and to the brainstem, basal forebrain, tuberomammillary nucleus, locus coeruleus, and thalamus (309). The cerebral cortex, including the prefrontal cortex, also projects to the basal forebrain, hypothalamus, and brainstem to further modulate arousal, wakefulness, and sleep (144). In addition, evidence implicates the striatum, parietal cortex, basal ganglia, ventromedial prefrontal cortex, nucleus accumbens, and anterior cingulate cortex in cognitive fatigue with brain injury (310). Evidence indicates that IL-1β can block orexin neuron activity in the lateral hypothalamus and that inflammation-induced lethargy is mediated by the suppression of orexin neuron activity (311).

## Vagus Nerve and CNS Inflammation

**Figure 2:**
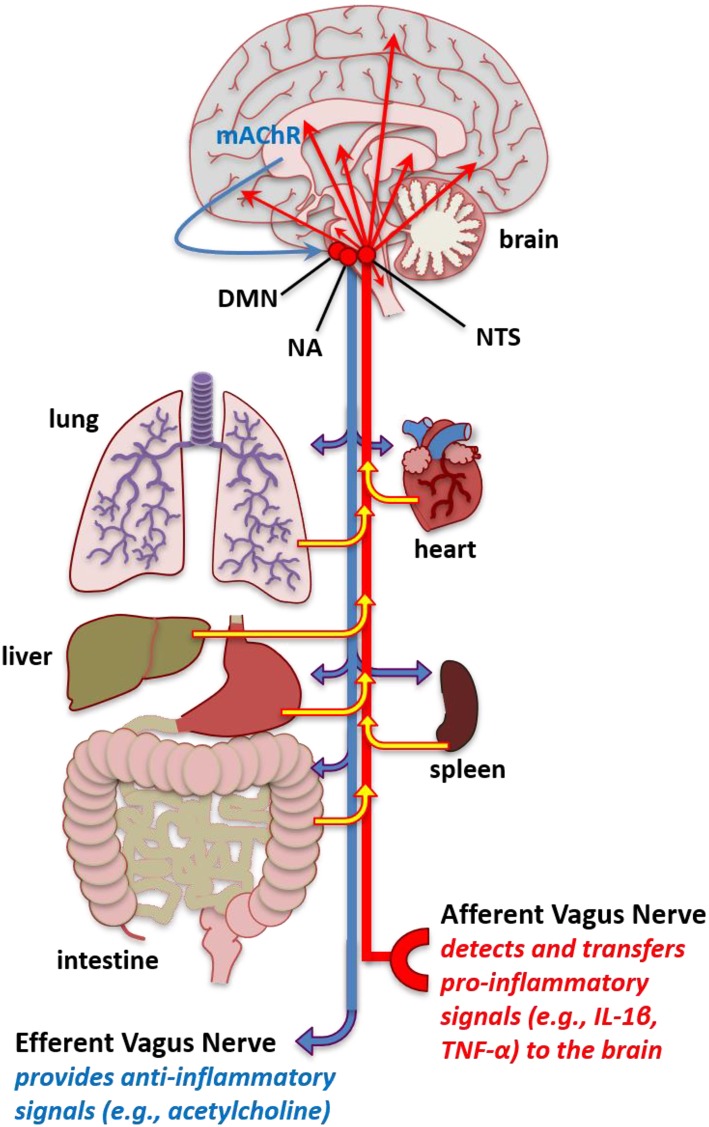
Increased pro-inflammatory molecules in the brain. Schematic of vagal afferent and efferent modulation of inflammation. The vagal afferents mediate pro-inflammatory signals, such as IL-1β and TNF-α from the periphery including the peritoneum and organs such as the lung intestine, hear, spleen, liver, and lung to stimulate inflammatory cytokines in the nucleus tractus solitarius (NTS). The NTS has projections to multiple brain areas where this pro-inflammatory signal that originates in the periphery leads to enhanced pro-inflammatory cytokine expression in brain areas that affect fatigue and sleep. Conversely, stimulation of the vagal efferents, such as that occurring from cholinergic mechanisms in the brain, such as muscarinic acetylcholine receptors (mAChR) acting through the, NTS, dorsal motor nucleus (DMN), and nucleus ambiguus (NA) can lead to anti-inflammatory reactions in peripheral tissues. The vagal afferents could serve to transfer enhanced inflammatory signals in the periphery occurring from autoimmune and related pathologies into dysregulated inflammatory regulation in the brain to induce fatigue-like behavior. Additionally, abnormal vagal efferent activity could server to affect physiological functions mediating fatigue-like behavior, such as heart rate, bronchoconstriction, and gluconeogenesis.

Two mechanisms whereby peripheral inflammation can enhance CNS inflammation are through leaky areas in the blood-brain-barrier and the vagus nerve (312). The vagus nerve, the tenth cranial nerve, is the longest nerve in the autonomic nervous system. This nerve has parasympathetic control of numerous organs that are involved in respiration—including the lungs, heart, and diaphragm—to mediate oxygen demand (313), which could contribute to fatigue. The vagal nerve afferents tend to relay pro-inflammatory responses cells from organs in the periphery to the CNS (Figure 2) (68). These vagal efferents tend to induce anti-inflammatory responses in peripheral tissue from CNS signals.

The vagal nerve afferents project to the dorsal vagal complex, which consists of the NTS, the dorsal motor nucleus (DMN) of the vagus, and the area postrema, located in the medulla area of the brainstem. However, the NTS is the primary area of vagal afferent stimulation. Neurons in the brainstem project toward many areas of the brain that can modulate fatigue including the amygdala, cortex, central nucleus of the amygdala, nucleus accumbens, paraventricular nucleus, and lateral hypothalamic areas of the hypothalamus, cerebellum, and other areas of the brainstem (314, 315). The NTS projects to areas of the brain that modulate the respiratory response (315), which could serve to alter oxygen and nutrient supply to affect fatigue. The NTS has projections to the other areas of the brain stem including the dorsal raphe nucleus and locus coeruleus. The NTS also projects to the parabrachial complex, which are nuclei located in the dorsolateral pons and surrounds the superior cerebellar peduncle. The parabrachial complex projects to areas of the brain involved in arousal including the thalamus, medial and lateral hypothalamus, and amygdala (316). Additionally, the NTS projects to the reticular formation, which are interconnected nuclei found thought the brainstem. The reticular formation has ascending pathways to the cortex in the ARAS (144).

Studies in rats and mice have demonstrated that the vagal afferent stimulation by IL-1β, TNF-α, or LPS delivered IP can enhance inflammatory IL-1β or TNF-α gene expression in the brain (69, 317, 318). Conversely, vagal efferents originating in the brainstem projecting to organs in the periphery can function to attenuate inflammation (68). Inhibiting the vagus nerve by vagotomy can demonstrate directional changes between the periphery and CNS. Inhibiting vagal afferents by a vagotomy and applying inflammatory substances to the periphery indicate that at lower dosages the vagal afferent nerves can be a major mechanism of translating peripheral inflammation to central inflammation and subsequently can affect behavior such as sleep (68, 69). Application of IL-1β, TNF-α, or LPS, which stimulates IL-1β and TNF-α production, into the peritoneum enhances sleep in mice and/or rats and is attenuated in animals that have their vagal nerves severed (69, 319, 320). Using vagotomy animal models, it has been shown that peripheral inflammation achieves this, in part, through stimulating the vagal afferents that enhance IL-1β and TNF-α in the NTS—a brain area that projects to brain areas involved in regulating sleep and are associated with modulating fatigue (69). In rats, the direct electrical stimulation of the afferent vagus nerve enhanced mRNA gene expression and protein levels in the hypothalamus and hippocampus (321). Nevertheless, the types of cytokines stimulating the vagal afferents from the periphery likely stimulate CNS cytokines over different time courses and affect different brain areas and cytokines. Thus, differences in peripheral inflammation associated with the type of autoimmune disease and progression of the disease could interact with circadian, sleep/wake states, activity-dependent, and pharmaceutical-related alterations in inflammatory processes to affect the severity or development of specific types of fatigue.

Vagotomy does not inhibit central inflammation induced by peripheral inflammation by large concentrations of inflammatory stimuli (69), suggesting that leaky areas of the blood-brain barrier also contribute to CNS inflammation. Leaky areas of the blood-brain-barrier exist in the circumventricular organs including the area postrema, and the subfornical organs, as well as the vascular organ of lamina terminalis (VOLT) (322). However, immunohistological evidence suggests that a diffusion barrier exists between the circumventricular organs and the NTS (323). VOLT, along with the subfornical organ, is interconnected with the mid-ventral hypothalamus and surrounds the third ventricle along with the median eminence. The VOLT capillaries do not have a blood-brain-barrier, and circulating factors present in the systemic circulation can enter the brain through this area (324). These areas are well-vascularized and respond to a wide variety of hormones and neurotransmitters. This area of cells heavily regulates osmoregulation, cardiovascular regulate, and energy homeostasis. Thus, leaky areas of the brainstem or the vagal afferents could be involved in peripheral inflammation enhancement in fatigue occurring in individuals with autoimmune disease that have enhanced peripheral inflammation.

The DMN and NTS are major sources of efferent motor vagal input. Longer preganglionic cholinergic neurons communicate with postganglionic neurons in closer proximity and within tissues of the viscera to induce anti-inflammatory signals (68). Acetylcholine released from neurons interacts with muscarinic acetylcholine receptors and utilizes the DMN, NTS, nucleus ambiguus (NA), and the vagal efferent nerve to alter heart rate, gluconeogenesis, and bronchial constriction (68). Consequently, these physiological effects could potentially affect fatigue-like behaviors.

Vagal efferent nerves project to the reticuloendothelial system, other peripheral organs, and the brain-derived motor output (325). Acetylcholine plays a large role in modulating the anti-inflammatory actions of the vagal efferent nerve on systemic and local peripheral inflammation (326). Muscarinic acetylcholine receptors in the CNS can induce anti-inflammatory effect on the periphery. This effect has been observed with the acetylcholinesterase inhibitor galantamine in the activation in the CNS. The nicotinic acetylcholine receptor alpha-7 (α7 nAChR) is a mechanism that signals the vagal efferent aspects of the anti-inflammatory effect. Cholinergic vagal efferent stimulation can downregulate CD14, TLR4, and NF-κB activation thus inhibiting pro-inflammatory processes within the periphery (68). This suppressor effect can be, in part, attributed to the activation of the JAK-STAT pathway. Additionally, the vagal efferents are involved in reducing hepatic glucose production and enhancement of glycogen synthesis and pancreas secretion of insulin, which could modulate metabolism and affect fatigue. Vagal nerve stimulation is used as an alternative therapy to inhibit TNF-α in patients with rheumatoid arthritis (327). Additional studies suggest that vagal nerve stimulation could benefit individuals with inflammatory bowel disease (327). The benefits of this anti-inflammatory treatment occur, in part, through the activation of cholinergic neurons to induce a suppression of peripheral inflammation, which then attenuates CNS inflammation. In fact, vagal nerve stimulation in individuals with Sjörgen's syndrome treated with a non-invasive method for 28 days were found to have reduced daytime sleepiness, improvements in fatigue, and reduced whole blood cells levels of TNF-α, IFN-γ, IL-6, and IL-1β (328).

Interestingly, evidence suggests relationships between peripheral muscle weakness and pain, inflammatory pathways, neuroinflammation, and fatigue in certain autoimmune disorders. A recent study found that serum TNF-related apoptosis-inducing ligand, which is a TNF-superfamily member, is enhanced in the serum and expressed in infiltrating inflammatory cells in patients with polymyostitis and dermatomyostitis (329)—conditions that are associated with increased fatigue and neuroinflammation. Chronic fatigue syndrome/myalgic encephalomyelitis is a condition that is defined by muscle pain and CNS inflammation, and fatigue (330) Thus, it is plausible that the vagus nerve is involved in muscle pain/CNS inflammation, and fatigue associated with these conditions. While much more research is needed to define the inflammatory pathways involved, targeting specific inflammatory pathways in the periphery could provide novel treatments to attenuate neuroinflammation and associated detriments.

## Neurovascular Unit and Vasohemodynamics

**Figure 3:**
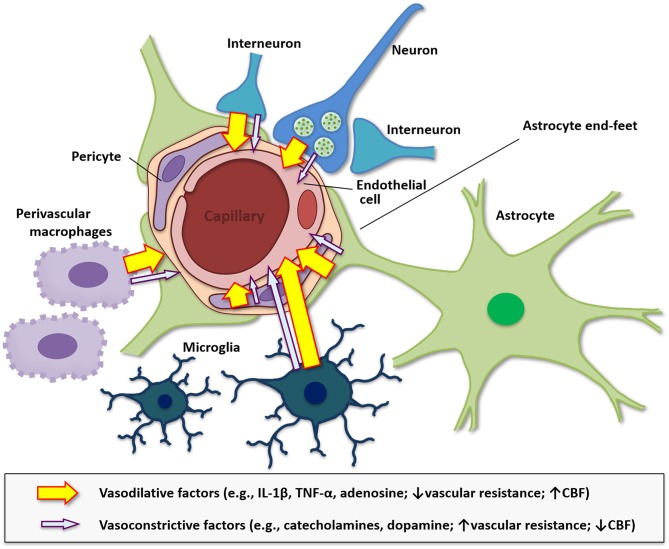
Diagram of the neurovascular unit in modulating vasohemodynamics. The neurovascular unit at the level of the cerebral microvasculature including the arterioles and capillaries is comprised of endothelial cells, smooth muscle, astrocytes, neurons, pericytes, and is modulated by surrounding microglia and perivascular macrophages. Additionally, alterations in metabolism and inflammation can modulate astrocyte end-feet to modulate cerebral blood flow (CBF). The neurovascular unit modulates blood flow throughout the brain and is regulated by energy needs of the surrounding cells and the vasoconstrictive, such as catecholamines and dopamine, and vasodilative factors, such as IL-1β, TNF-α, and adenosine, that are released by these cells. Pro-inflammatory molecules tend to be vasodilative, reduce vascular resistance, and increase cerebral CBF, while monoamines released by neurons have both vasodilative and vasoconstrictive properties, which can influence blood flow. Vasoconstrictive substances typically increase vascular resistance and reduce CBF.

Cerebral blood flow is an essential component involved in providing nutrients and oxygen supply to cells in the CNS (331). Additionally, the neurovasculature removes waste products including carbon dioxide (CO_2_), signaling molecules, and provides adequate supplies of energy reserves (332, 333). A reduction in blood supply within an area surrounding neurons in the CNS can lead to cellular and performance dysfunction (334). Autoimmune diseases, such as multiple sclerosis, type 1 diabetes, and Alzheimer's disease, and celiac disease are often found to be associated with hypoperfusion (335–337) Neural activity functions in synchrony with localized cerebral blood flow and is referred to as neurovascular coupling (338). This neural activity is associated with increases in the release of vasoactive substances, which can directly or indirectly alter vasohemodynamics (339). Pro-inflammatory cytokines including IL-1β and TNF-α are reported alter vasohemodynamics, although these relationships are not well-understood (340–343). In rats, IL-1β applied ICV induced global cerebral hypoperfusion (340). In rabbits, application of TNF-α into the cisterna magna induces a prolonged reduction in cerebral blood flow (343). It has been postulated under the hemo-neuro hypothesis that vasohemodynamics modulate the gain of local cortical circuits to alter the detection and discrimination of sensory stimuli, and thus, cerebral blood flow could affect neural activity (344). This process could involve alterations in diffusible or mechanical factors that lead to alterations in neuronal membranes, temperature, or indirect mechanisms such as altering astrocyte functions, which are tightly regulated with neurons. Indeed, neurovascular coupling depends not only on neurons but also numerous cells of the neurovascular unit such as neurons, astrocytes with end feet that surround the vasculature, endothelial cells, pericytes, and surrounding supporting cells including microglia and perivascular macrophages (Figure 3) (338, 345, 346). Interestingly, inhibiting perivascular macrophages and brain macrophages using liposomes with clodronate can reduce IL-1β in the brain and increase central mediated fatigue in an exhaustive exercise test in mice (347), which suggests that inflammation in the neurovascular unit is critical to CNS-mediated fatigue.

The neurovascular unit functions to regulate cerebral blood flow, blood velocity, and blood volume in local areas of the brain (333, 338). Substances can pass through blood capillaries by osmosis, filtration, or diffusion. Oxygen and CO_2_ pass through the blood vessel walls by diffusion whereas fluid passes through by both hydrostatic and osmotic pressure (348). Blood travels from areas of high pressure to areas of lower pressure and flows in the direction of the lower pressure gradient (349). Blood velocity changes inversely with the cross-sectional area of the blood vessels, where vasodilation will increase blood flow and blood volume (350). Blood pressure can affect blood flow in the circulation including the cerebral vasculature (351). Blood pressure is affected by cardiac output, blood volume, peripheral resistance, and viscosity, which is dependent on the heart and the lung. Systemic blood pressure is expressed as the ratio of systolic pressure to diastolic pressure. Cerebral autoregulation is the process where cerebrovascular resistance, which is largely affected by the diameter of blood vessels, is adjusted to compensate for changes in perfusion pressure to maintain a constant blood flow. The mean arterial pressure is the average pressure of blood in the arteries driving blood into vessels and is dependent upon systolic and diastolic blood pressure values.

There is evidence that autoimmune conditions are associated with increased hypertension including systemic lupus erythematosus, psoriasis, multiple sclerosis, and rheumatoid arthritis (352, 353). Inflammation is a primary contributor to hypertension (354). Hypertension is associated with injured endothelium, which affects microvessel integrity. Astrocyte end feet interact with the endothelium to form the basal lamina matrix, which forms a blood-brain-barrier by forming tight junctions that inhibit larger molecules, including pro-inflammatory cytokines, from entering the CNS (355). Prolonged enhanced inflammation can make the vascular more rigid and less compliant (356). Vascular compliance is a necessary process that allows the vasculature to dilate or contract appropriately when required by an activity, and the lack of proper function could result in fatigue. A major component in modulating blood flow is vascular compliance, which allows increased blood volume to occur and resultant increases in blood flow. Impairments in vessel compliance are seen in inflammatory conditions such as cardiovascular disease and autoimmune diseases such as multiple sclerosis, type 1 diabetes, and systemic lupus erythematosus (357–360). Moreover, pro-inflammatory cytokines, such as TNF-α, are elevated with reductions in vascular compliance in patients with systemic lupus erthematosus (358).

Multiple autoimmune diseases, such as rheumatoid arthritis, Sjörgen's syndrome (SS), Wegener's granulomatosis, Churg-Strauss syndrome, Good pasture's syndrome, and ankylosing spondylitis are known to induce inflammation in the lung and alter lung functions (361). Macrophages in the lungs are located in the airways, alveoli, lung interstitium and can travel into the lung microvasculature (362). These cells are potent sources of cytokines, chemokines, and other pro-inflammatory molecules (363). Pulmonary hemodynamics with associated impairments in pulmonary vascular compliance and increased vascular resistance can persist following enhancements in exercise in individuals with pulmonary hypertension suggesting that lung function is important in fatigue (364).

Hyperventilation can induce fatigue and is implicated in perpetuating chronic fatigue syndrome/myalgic encephalomyelitis (365). A condition related to chronic fatigue syndrome/myalgic encephalomyelitis, postural osrthostatic tachycardia syndrome, is a conditions that induces cerebrovasoconstriction and hyperventilation affecting cerebral blood flow (366, 367). Interestingly, CNS related deficits, such as cognitive impairments are found in individuals with chronic fatigue syndrome and postural orthostatic tachycardia syndrome in support of the idea that central-mediated impairments could be, in part, induced from hyperventilation (368). Conditions of dysfunctional breathing, such as asthma and chronic obstructive pulmonary disease (369), are often associated with hyperventilation during exercise. Thus, it would be expected that individuals with dyspnea, such as those with chronic fatigue syndrome/myalgic encephalomyelitis, could have exercise-induced hyperventilation and associated fatigue.

A complex and not well-understood relationship exists between the brain vasculature, cerebrospinal fluid, and the perivascular spaces that act as lymphatics in the brain (30). In 1968, Foldi and colleagues alluded that perivascular spaces function as lymphatics in the brain (370), which has since been termed the glymphatic system (371). They glymphatic system is modulated by cerebrovascular pulsation and astrocyte water channels around the perivascular channels. The glymphatic system is altered during sleep/wake periods and is thought to clear the formation of ROS and other waste products in the brain. Persistent inflammation can alter astrocytic channels and end-feet morphology (372), which could modulate the clearance and lead to further inflammation affecting fatigue and/or sleep. Although speculative, it is plausible that an enhanced ROS content in the CNS might be unable to be cleared in autoimmune diseases and sleep related disorders leading to exacerbated inflammation and impaired vasohemodynamics leading to fatigue.

## Future Directions

There are several areas of research and logistics that need to be established to understand the exact mechanisms of fatigue and sleep in autoimmune diseases. First, a detailed description of particular types of fatigue needs to be established in the clinic. This can be achieved, in part, with more precise medical coding. Second, there needs to be standardized questionnaires and diagnostic tests that can more precisely indicate the determents observed from fatigue. This will provide insight into the types of fatigue that are observed with the pathogenesis of the autoimmune disorders. Understanding the neurocircuitry of fatigue and its relationship between inflammation and autoimmune diseases is also needed. This area can aid in understanding the manifestation of types and severity of fatigue found in autoimmune patients. Since inflammation and metabolism are implicated in fatigue and impairments are found in these in autoimmune disorders, a detailed understating of the mechanisms of these systems, unique cells, and brain areas are lacking in autoimmune research. More information on interactions between circadian timing and sleep/wake state or sleep loss, alterations in neuronal activity, and normal daily functioning that affect fatigue is required. And there needs to be a better understanding of the relationship between the vagal afferents in modulating brain inflammation to induce fatigue is needed. More research is also necessary for understanding the relationship between vasohemodynamics and fatigue in autoimmune disease. Additionally, several autoimmune diseases are associated with disproportionately greater incidence and disease severity in particular genders and little information is known regarding the relationship of fatigue with these gender differences (373). The relationship of gender to fatigue in autoimmune disease should be a topic for future research.

An autoimmune disease involves multiple interactions between genetics and environmental factors. Most autoimmune-related disorders are more prevalent in monozygotic twins vs. dizygotic twins or siblings, indicating the involvement of genetics in disease development. Furthermore, genome wide association studies (GWAS) have found several genetic loci and small nucleotide polymorphisms that are associated with specific autoimmune disease prevalence. Interestingly, proteins encoded by genes that are involved in inflammatory mechanisms including NF-κB, apoptosis, Toll-like receptor, and immune complexes are described in autoimmune and related disorders. Nevertheless, a number of autoimmune diseases exhibit similar genetic modifications, which likely contributes to the higher incidence of multiple autoimmune diseases found in individuals with autoimmune disorders and potentially similar disease characteristics including fatigue. Indeed, larger data sets (i.e., >10,000 individuals) are finding new associations of inflammatory mechanism I with impairments in respiratory function and sleep (374). Several inflammatory gene small nucleotide polymorphisms have been implicated in fatigue including TNF-α, IL-1β, IL-6, and IFN-γ (375). Nevertheless, a high amount of the heritability of the genetics behind autoimmune and related disorders remains unexplained. In recent years, researchers, agencies, and governments, such as the British Biobank, Trans-Omics for Precision Medicine (TOPMED), and the Million Veteran Program have begun assembling very large sample populations that are giving enough statistical power to unmask genetic links between diseases.

## Conclusion

In summary, fatigue is a major early finding in individuals with autoimmune diseases, and inflammation is a contributing factor relating to this impairment, one that affects the ability of people to perform daily activities, work, and thus their overall well-being. Recent research reveals a relationship between types of fatigue and certain brain areas, cell types, and phenotypes that mediate the symptoms observed. In addition, inflammatory molecules that are enhanced in the periphery with specific types of autoimmune disease can alter brain inflammation and neurocircuitry affecting fatigue. Consequently, immunomodulatory agents and drugs targeting inflammatory pathways could serve to treat fatigue occurring in autoimmune and related diseases. Understanding the mechanisms behind fatigue will not only aid individuals with autoimmune diseases but could also benefit transplant recipients, cancer patients, and infectious disease patients who experience debilitating fatigue.

## Author Contributions

All authors listed have made a substantial, direct and intellectual contribution to the work, and approved it for publication.

### Conflict of Interest Statement

The authors declare that the research was conducted in the absence of any commercial or financial relationships that could be construed as a potential conflict of interest.

## Abbreviations

5-HT: 5-hydroxytryptamine
ACTH: adrenocorticotropic hormone
AIM2: absent in melanoma 2
AKT: protein kinase B
AMPA: alpha-amino-3-hydroxy-5-methyl-4-isoxazolepropionic acid
AP-1: activator protein-1
ARAS: ascending reticular activating system
ASC: adapter apoptosis-associated speck-like protein containing a C-terminal caspase recruitment domain
ATP: adenosine triphosphate
BMAL1: brain and muscle aryl hydrocarbon receptor nuclear translocator-like protein 1
CBF: cerebral blood flow
CD39: adenosine monophosphate by ectonucleoside diphosphohydrolase
CD73: adenosine by ecto-5' nucleotidase
cGAS: cyclic guanosine monophosphate-adenosine monophosphate synthase
CLK: CLOCK
CNS: central nervous system
CO_2_: carbon dioxide
CREB: cyclic AMP response element-binding protein
CRH: corticotrophin-releasing hormone
CYC: CYCLE
DAMPS: danger-associated molecular patterns
DNA: single-stranded deoxyribonucleic acid
EAE: experimental autoimmune encephalomyelitis
ERK: extracellular signal regulated kinase
GABA: gamma-aminobutyric acid
GWAS: genome wide association studies
HIV-1: Human Immunodeficiency Virus-1
HPA: hypothalamic-pituitary-adrenal
ICV: intracerebroventricular
IFN-γ: interferon-gamma
IκB: inhibitory kappa B
IKK: inhibitor of kappa B kinase
IL-1: interleukin
IL-1β: Interleukin-1 beta
IL-1R1: IL-1 receptor type I
IL-1RA: IL-1 receptor antagonist
IL-1RAP: IL-1 receptor accessory protein
IP: intraperitoneal
IRAK4: interleukin-1 receptor associated kinase 4
IRF3: interferon regulatory factor 3
JAK: Janus kinase
JNK: c-Jun N-terminal nucleated kinase
LPS: lipopolysaccharide
MAPK: mitogen-activated protein kinase
MAPKK: mitogen-activated protein kinase kinase
mTOR: mammalian target of the rapamycin
mTORC: mechanistic target of rapamycin
MYD88: myeloid differentiation primary response 88
NAC: N-acetylcysteine
NADPH: nicotinamide adenine dinucleotide phosphate
α7 nAChR: nicotinic acetylcholine receptor alpha-7
NF-κB: nuclear factor kappa B
NLRP3: nucleotide leucine-rich protein-3
NREM: non-rapid-eye movement
NTS: nucleus tractus solitarii
OXPHOS: oxidative phosphorylation
P2: purine type 2
P2X7: purine type 2 X7
PAMPS: pathogen-associated molecular patterns
PER: PERIOD
PI3-K: phosphatidylinositol 3-kinase
PPP: pentose phosphate pathway
PRR: pattern recognition receptor
REM: rapid-eye movement
RNA: ribonucleic acid
ROS: reactive oxygen species
SOCS1: suppressor of cytokine signaling 1
SS: Sjörgen's syndrome
STAT: signal transducer and activator of transcription protein
STING: stimulator of interferon genes
TGF-β: transforming growth factor-beta
TLR4: toll-like receptor 4
TNF-α: tumor necrosis factor-alpha
TOPMED: trans-Omics for Precision Medicine
TRX: Thioredoxin
TXNIP: thioredoxin-interactive protein
VOLT: vascular organ of lamina terminalis.

## References

[1] American Autoimmune Related Disease Association Autoimmune Disease Statistics AARDA. Available online at: https://www.aarda.org/news-information/statistics/ (accessed March 17, 2019).

[2] Diseases, & Conditions|NIH: National Institute of Allergy and Infectious Diseases. Available online at: https://www.niaid.nih.gov/diseases-conditions/all (accessed March 24, 2019).

[3] Autoimmune Disease List • AARDA. Available online at: https://www.aarda.org/diseaselist/ (accessed March 17, 2019).

[4] Bakker JP Weaver TE Parthasarathy S Aloia MS. Adherence to CPAP: what should we be aiming for, and how can we get there? Chest. (2019) 155:1272–87. 10.1016/j.chest.2019.01.012 30684472

[5] Lerner A Jeremias P Matthias T The world incidence and prevalence of autoimmune diseases is increasing. Int J Celiac Dis. (2016) 3:151–5. 10.12691/ijcd-3-4-8

[6] Fatigue Survey Results Released • AARDA. Available online at: https://www.aarda.org/fatigue-survey-results-released/ (accessed March 17, 2019).

[7] Chalah MA Ayache SS. Is there a link between inflammation and fatigue in multiple sclerosis? J Inflamm Res. (2018) 11:253–64. 10.2147/JIR.S167199 29922081 PMC5995280

[8] Griggs S Morris NS. Fatigue among adults with type 1 diabetes mellitus and implications for self-management: an integrative review. Diabetes Educ. (2018) 44:325–39. 10.1177/0145721718782148 29944065 PMC6372920

[9] Torres J Mehandru S Colombel J-F Peyrin-Biroulet L. Crohn's disease. Lancet. (2017) 389:1741–55. 10.1016/S0140-6736(16)31711-1 27914655

[10] Nguyen MH Bryant K O'Neill SG. Vitamin D in SLE: a role in pathogenesis and fatigue? A review of the literature. Lupus. (2018) 27:2003–11. 10.1177/0961203318796293 30157716

[11] Hajjar J Kutac C Rider NL Seeborg FO Scalchunes C Orange J. Fatigue and the wear-off effect in adult patients with common variable immunodeficiency. Clin Exp Immunol. (2018) 194:327–38. 10.1111/cei.13210 30168848 PMC6231022

[12] Hajjar J Guffey D Minard CG Orange JS. Increased incidence of fatigue in patients with primary immunodeficiency disorders: prevalence and associations within the US immunodeficiency network registry. J Clin Immunol. (2017) 37:153–65. 10.1007/s10875-016-0367-1 28124237 PMC5326589

[13] Junghaenel DU Christodoulou C Lai J-S Stone AA. Demographic correlates of fatigue in the US general population: results from the patient-reported outcomes measurement information system (PROMIS) initiative. J Psychosom Res. (2011) 71:117–23. 10.1016/j.jpsychores.2011.04.007 21843744 PMC3744100

[14] Enns MW Bernstein CN Kroeker K Graff L Walker JR Lix LM. The association of fatigue, pain, depression and anxiety with work and activity impairment in immune mediated inflammatory diseases. PLoS ONE. (2018) 13:e0198975. 10.1371/journal.pone.0198975 29879231 PMC5991721

[15] Aghaei N Karbandi S Gorji MAH Golkhatmi MB Alizadeh B. Social support in relation to fatigue symptoms among patients with multiple sclerosis. Indian J Palliat Care. (2016) 22:163–7. 10.4103/0973-1075.179610 27162427 PMC4843555

[16] Flachenecker P. Autoimmune diseases and rehabilitation. Autoimmun Rev. (2012) 11:219–25. 10.1016/j.autrev.2011.05.016 21619946

[17] Cook KF Molton IR Jensen MP. Fatigue and aging with a disability. Arch Phys Med Rehabil. (2011) 92:1126–33. 10.1016/j.apmr.2011.02.017 21704793

[18] Agarwal N Kumar V. Burden of lupus on work: issues in the employment of individuals with lupus. Work. (2016) 55:429–39. 10.3233/WOR-162398 27689581

[19] Finsterer J Mahjoub SZ. Fatigue in healthy and diseased individuals. Am J Hosp Palliat Care. (2014) 31:562–75. 10.1177/1049909113494748 23892338

[20] Taylor PC Moore A Vasilescu R Alvir J Tarallo M. A structured literature review of the burden of illness and unmet needs in patients with rheumatoid arthritis: a current perspective. Rheumatol Int. (2016) 36:685–95. 10.1007/s00296-015-3415-x 26746843 PMC4839053

[21] Sabes-Figuera R McCrone P Hurley M King M Donaldson AN Ridsdale L. The hidden cost of chronic fatigue to patients and their families. BMC Health Serv Res. (2010) 10:56. 10.1186/1472-6963-10-56 20202216 PMC2845126

[22] Boissoneault J Letzen J Robinson M Staud R. Cerebral blood flow and heart rate variability predict fatigue severity in patients with chronic fatigue syndrome. Brain Imaging Behav. (2018) 13:789–97. 10.1007/s11682-018-9897-x 29855991 PMC6274602

[23] Trinity JD Broxterman RM Richardson RS. Regulation of exercise blood flow: role of free radicals. Free Radic Biol Med. (2016) 98:90–102. 10.1016/j.freeradbiomed.2016.01.017 26876648 PMC4975999

[24] Lucas K Maes M. Role of the Toll Like receptor (TLR) radical cycle in chronic inflammation: possible treatments targeting the TLR4 pathway. Mol Neurobiol. (2013) 48:190–204. 10.1007/s12035-013-8425-7 23436141 PMC7091222

[25] Dantzer R Heijnen CJ Kavelaars A Laye S Capuron L. The neuroimmune basis of fatigue. Trends Neurosci. (2014) 37:39–46. 10.1016/j.tins.2013.10.003 24239063 PMC3889707

[26] Popp RFJ Fierlbeck AK Knüttel H König N Rupprecht R Weissert R. Daytime sleepiness versus fatigue in patients with multiple sclerosis: a systematic review on the Epworth sleepiness scale as an assessment tool. Sleep Med Rev. (2017) 32:95–108. 10.1016/j.smrv.2016.03.004 27107751

[27] Nociti V Losavio FA Gnoni V Losurdo A Testani E Vollono C. Sleep and fatigue in multiple sclerosis: a questionnaire-based, cross-sectional, cohort study. J Neurol Sci. (2017) 372:387–92. 10.1016/j.jns.2016.10.040 27823835

[28] Moulton CD Pavlidis P Norton C Norton S Pariante C Hayee B. Depressive symptoms in inflammatory bowel disease: an extraintestinal manifestation of inflammation? Clin Exp Immunol. (2019). [Epub ahead of print]. 10.1111/cei.13276 30762873 PMC6693970

[29] Katarina V Gordana T Svetlana MD Milica B. Oxidative stress and neuroinflammation should be both considered in the occurrence of fatigue and depression in multiple sclerosis. Acta Neurol Belg. (2018). [Epub ahead of print]. 10.1007/s13760-018-1015-8 30182258

[30] Zielinski MR McKenna JT McCarley RW. Functions and mechanisms of sleep. AIMS Neurosci. (2016) 3:67–104. 10.3934/Neuroscience.2016.1.67 28413828 PMC5390528

[31] Lacourt TE Vichaya EG Chiu GS Dantzer R Heijnen CJ. The high costs of low-grade inflammation: persistent fatigue as a consequence of reduced cellular-energy availability and non-adaptive energy expenditure. Front Behav Neurosci. (2018) 12:78. 10.3389/fnbeh.2018.00078 29755330 PMC5932180

[32] Atkins C Wilson AM. Managing fatigue in sarcoidosis - A systematic review of the evidence. Chron Respir Dis. (2017) 14:161–73. 10.1177/1479972316661926 27507833 PMC5720218

[33] Duarte-Delgado NP Vásquez G Ortiz-Reyes BL. Blood-brain barrier disruption and neuroinflammation as pathophysiological mechanisms of the diffuse manifestations of neuropsychiatric systemic lupus erythematosus. Autoimmun Rev. (2019) 18:426–32. 10.1016/j.autrev.2018.12.004 30763633

[34] Voet S Prinz M van Loo G. Microglia in central nervous system inflammation and multiple sclerosis pathology. Trends Mol Med. (2019) 25:112–23. 10.1016/j.molmed.2018.11.005 30578090

[35] Luty J Ruckemann-Dziurdzinska K Witkowski JM Bryl E. Immunological aspects of autoimmune thyroid disease - Complex interplay between cells and cytokines. Cytokine. (2019) 116:128–33. 10.1016/j.cyto.2019.01.003 30711852

[36] Toubi E Vadasz Z. Innate immune-responses and their role in driving autoimmunity. Autoimmun Rev. (2019) 18:306–11. 10.1016/j.autrev.2018.10.005 30639645

[37] Salvi V Gianello V Tiberio L Sozzani S Bosisio D. Cytokine targeting by miRNAs in autoimmune diseases. Front Immunol. (2019) 10:15. 10.3389/fimmu.2019.00015 30761124 PMC6361839

[38] Santos JC Pyter LM. Neuroimmunology of behavioral comorbidities associated with cancer and cancer treatments. Front Immunol. (2018) 9:1195. 10.3389/fimmu.2018.01195 29930550 PMC6001368

[39] Vinnikov D Blanc PD Alilin A Zutler M Holty J-EC. Fatigue and sleepiness determine respiratory quality of life among veterans evaluated for sleep apnea. Health Qual Life Outcomes. (2017) 15:48. 10.1186/s12955-017-0624-x 28288646 PMC5348814

[40] Rosenzweig I Williams SCR Morrell MJ. The impact of sleep and hypoxia on the brain: potential mechanisms for the effects of obstructive sleep apnea. Curr Opin Pulm Med. (2014) 20:565–71. 10.1097/MCP.0000000000000099 25188719

[41] Cumming TB Packer M Kramer SF English C. The prevalence of fatigue after stroke: a systematic review and meta-analysis. Int J Stroke. (2016) 11:968–77. 10.1177/1747493016669861 27703065

[42] Barrington J Lemarchand E Allan SM. A brain in flame; do inflammasomes and pyroptosis influence stroke pathology? Brain Pathol. (2017) 27:205–12. 10.1111/bpa.12476 27997059 PMC8028888

[43] Cronin H O'Loughlin E. Sleep and fatigue after TBI. NeuroRehabilitation. (2018) 43:307–17. 10.3233/NRE-182484 30347625

[44] Mortezaee K Khanlarkhani N Beyer C Zendedel A. Inflammasome: its role in traumatic brain and spinal cord injury. J Cell Physiol. (2018) 233:5160–9. 10.1002/jcp.26287 29150951

[45] Cincotta MC Engelhard MM Stankey M Goldman MD. Fatigue and fluid hydration status in multiple sclerosis: a hypothesis. Mult Scler. (2016) 22:1438–43. 10.1177/1352458516663854 27542703 PMC5156578

[46] Louati K Berenbaum F. Fatigue in chronic inflammation - a link to pain pathways. Arthritis Res Ther. (2015) 17:254. 10.1186/s13075-015-0784-1 26435495 PMC4593220

[47] Katz P. Causes and consequences of fatigue in rheumatoid arthritis. Curr Opin Rheumatol. (2017) 29:269–76. 10.1097/BOR.0000000000000376 28207494

[48] Theofilidis G Bogdanis GC Koutedakis Y Karatzaferi C. Monitoring exercise-induced muscle fatigue and adaptations: making sense of popular or emerging indices and biomarkers. Sport. (2018) 6:153. 10.3390/sports6040153 30486243 PMC6315493

[49] Antonelli A Fallahi P Di Bari F Giuggioli D Ferrari SM Ferri C. Fatigue in patients with systemic sclerosis and hypothyroidism. A review of the literature and report of our experience. Clin Exp Rheumatol. (2019). 35(Suppl. 106):193–7. 28375832

[50] Wang XS Woodruff JF. Cancer-related and treatment-related fatigue. Gynecol Oncol. (2015) 136:446–52. 10.1016/j.ygyno.2014.10.013 25458588 PMC4355326

[51] Burnley M Jones AM. Power-duration relationship: physiology, fatigue, and the limits of human performance. Eur J Sport Sci. (2018) 18:1–12. 10.1080/17461391.2016.1249524 27806677

[52] Boccia G Dardanello D Rinaldo N Coratella G Schena F Rainoldi A. Electromyographic manifestations of fatigue correlate with pulmonary function, 6-minute walk test, and time to exhaustion in COPD. Respir Care. (2015) 60:1295–302. 10.4187/respcare.04138 26286735

[53] Felger JC Treadway MT. Inflammation effects on motivation and motor activity: role of dopamine. Neuropsychopharmacology. (2017) 42:216–41. 10.1038/npp.2016.143 27480574 PMC5143486

[54] Pollmächer T Schuld A Kraus T Haack M Hinze-Selch D Mullington J. Experimental immunomodulation, sleep, and sleepiness in humans. Ann NY Acad Sci. (2000) 917:488–99. 10.1111/j.1749-6632.2000.tb05413.x 11268376

[55] Mullington JM Hinze-Selch D Pollmächer T. Mediators of inflammation and their interaction with sleep: relevance for chronic fatigue syndrome and related conditions. Ann NY Acad Sci. (2001) 933:201–10. 10.1111/j.1749-6632.2001.tb05825.x 12000021

[56] Rizzo FR Musella A De Vito F Fresegna D Bullitta S Vanni V. Tumor necrosis factor and interleukin-1β modulate synaptic plasticity during neuroinflammation. Neural Plast. (2018) 2018:8430123. 10.1155/2018/8430123 29861718 PMC5976900

[57] Renna ME O'Toole MS Spaeth PE Lekander M Mennin DS. The association between anxiety, traumatic stress, and obsessive-compulsive disorders and chronic inflammation: a systematic review and meta-analysis. Depress Anxiety. (2018) 35:1081–94. 10.1002/da.22790 30199144

[58] Jeon SW Kim Y-K. Inflammation-induced depression: Its pathophysiology and therapeutic implications. J Neuroimmunol. (2017) 313:92–8. 10.1016/j.jneuroim.2017.10.016 29153615

[59] Konsman JP Inflammation and depression: a nervous plea for psychiatry to not become immune to interpretation. Pharmaceuticals. (2019) 12:29 10.3390/ph12010029 PMC6469164 30769887

[60] Deak T Quinn M Cidlowski JA Victoria NC Murphy AZ Sheridan JF. Neuroimmune mechanisms of stress: sex differences, developmental plasticity, and implications for pharmacotherapy of stress-related disease. Stress. (2015) 18:367–80. 10.3109/10253890.2015.1053451 26176590 PMC4813310

[61] Murphy K Kenneth M Weaver C Janeway's Immunobiology. 9th Edn New York, NY: Garland Science (2017).

[62] Galic MA Riazi K Pittman QJ. Cytokines and brain excitability. Front Neuroendocrinol. (2012) 33:116–25. 10.1016/j.yfrne.2011.12.002 22214786 PMC3547977

[63] Morris G Berk M Walder K Maes M. Central pathways causing fatigue in neuro-inflammatory and autoimmune illnesses. BMC Med. (2015) 13:28. 10.1186/s12916-014-0259-2 25856766 PMC4320458

[64] Kuwabara T Ishikawa F Kondo M Kakiuchi T. The role of IL-17 and related cytokines in inflammatory autoimmune diseases. Mediators Inflamm. (2017) 2017:3908061. 10.1155/2017/3908061 28316374 PMC5337858

[65] Taniyama K Kuno T Tanaka C. Distribution of beta-adrenoceptors associated with cAMP-generating system in cat colon. Am J Physiol. (1987) 253(3 Pt 1):G378–82. 10.1152/ajpgi.1987.253.3.G378 2820239

[66] Hilhorst M Shirai T Berry G Goronzy JJ Weyand CM. T cell-macrophage interactions and granuloma formation in vasculitis. Front Immunol. (2014) 5:432. 10.3389/fimmu.2014.00432 25309534 PMC4162471

[67] Funes SC Rios M Escobar-Vera J Kalergis AM. Implications of macrophage polarization in autoimmunity. Immunology. (2018) 154:186–95. 10.1111/imm.12910 29455468 PMC5980179

[68] Pavlov VA Tracey KJ. The vagus nerve and the inflammatory reflex–linking immunity and metabolism. Nat Rev Endocrinol. (2012) 8:743–54. 10.1038/nrendo.2012.189 23169440 PMC4082307

[69] Zielinski MR Dunbrasky DL Taishi P Souza G Krueger JM. Vagotomy attenuates brain cytokines and sleep induced by peripherally administered tumor necrosis factor-α and lipopolysaccharide in mice. Sleep. (2013) 36:1227–38. 10.5665/sleep.2892 23904683 PMC3700720

[70] Zielinski MR Krueger JM. Sleep and innate immunity. Front Biosci. (2011) 3:632–42. 10.2741/s176 21196401 PMC3645929

[71] Hodes GE Ménard C Russo SJ. Integrating Interleukin-6 into depression diagnosis and treatment. Neurobiol Stress. (2016) 4:15–22. 10.1016/j.ynstr.2016.03.003 27981186 PMC5146277

[72] Morris G Berk M Galecki P Walder K Maes M. The neuro-immune pathophysiology of central and peripheral fatigue in systemic immune-inflammatory and neuro-immune diseases. Mol Neurobiol. (2016) 53:1195–219. 10.1007/s12035-015-9090-9 25598355

[73] Zhao R Zhou H Su SB. A critical role for interleukin-1β in the progression of autoimmune diseases. Int Immunopharmacol. (2013) 17:658–69. 10.1016/j.intimp.2013.08.012 24012439

[74] Yadlapati S Efthimiou P. Impact of IL-1 inhibition on fatigue associated with autoinflammatory syndromes. Mod Rheumatol. (2016) 26:3–8. 10.3109/14397595.2015.1069459 26140469

[75] Grossberg AJ Vichaya EG Christian DL Molkentine JM Vermeer DW Gross PS. Tumor-associated fatigue in cancer patients develops independently of IL1 signaling. Cancer Res. (2018) 78:695–705. 10.1158/0008-5472.CAN-17-2168 29217760 PMC5811314

[76] Dinarello CA. Overview of the IL-1 family in innate inflammation and acquired immunity. Immunol Rev. (2018) 281:8–27. 10.1111/imr.12621 29247995 PMC5756628

[77] Patra MC Choi S. Recent progress in the molecular recognition and therapeutic importance of interleukin-1 receptor-associated kinase 4. Molecules. (2016) 21:1529. 10.3390/molecules21111529 27845762 PMC6274160

[78] Bonney EA. Mapping out p38MAPK. Am J Reprod Immunol. (2017) 77:e12652. 10.1111/aji.12652 28194826 PMC5527295

[79] Kappelmann M Bosserhoff A Kuphal S. AP-1/c-Jun transcription factors: regulation and function in malignant melanoma. Eur J Cell Biol. (2014) 93:76–81. 10.1016/j.ejcb.2013.10.003 24315690

[80] Boraschi D Italiani P Weil S Martin MU. The family of the interleukin-1 receptors. Immunol Rev. (2018) 281:197–232. 10.1111/imr.12606 29248002

[81] Smith DE Lipsky BP Russell C Ketchem RR Kirchner J Hensley K. A central nervous system-restricted isoform of the interleukin-1 receptor accessory protein modulates neuronal responses to interleukin-1. Immunity. (2009) 30:817–31. 10.1016/j.immuni.2009.03.020 19481478 PMC4103746

[82] Taishi P Davis CJ Bayomy O Zielinski MR Liao F Clinton JM. Brain-specific interleukin-1 receptor accessory protein in sleep regulation. J Appl Physiol. (2012) 112:1015–22. 10.1152/japplphysiol.01307.2011 22174404 PMC3311656

[83] Borragán G Gilson M Atas A Slama H Lysandropoulos A De Schepper M. Cognitive fatigue, sleep and cortical activity in multiple sclerosis disease. A behavioral, polysomnographic and functional near-infrared spectroscopy investigation. Front Hum Neurosci. (2018) 12:378. 10.3389/fnhum.2018.00378 30294266 PMC6158319

[84] Huang Z-B Sheng G-Q. Interleukin-1β with learning and memory. Neurosci Bull. (2010) 26:455–68. 10.1007/s12264-010-6023-5 21113196 PMC5560336

[85] O'Léime CS Cryan JF Nolan YM. Nuclear deterrents: Intrinsic regulators of IL-1β-induced effects on hippocampal neurogenesis. Brain Behav Immun. (2017) 66:394–412. 10.1016/j.bbi.2017.07.153 28751020

[86] Ferezou I Bolea S Petersen CCH. Visualizing the cortical representation of whisker touch: voltage-sensitive dye imaging in freely moving mice. Neuron. (2006) 50:617–29. 10.1016/j.neuron.2006.03.043 16701211

[87] Shine JM Breakspear M Bell PT Ehgoetz Martens KA Shine R Koyejo O Human cognition involves the dynamic integration of neural activity and neuromodulatory systems. Nat Neurosci. (2019) 22:289–96. 10.1038/s41593-018-0312-0 30664771

[88] Hallett H Churchill L Taishi P De A Krueger JM. Whisker stimulation increases expression of nerve growth factor- and interleukin-1beta-immunoreactivity in the rat somatosensory cortex. Brain Res. (2010) 1333:48–56. 10.1016/j.brainres.2010.03.048 20338152 PMC2879054

[89] Churchill L Rector DM Yasuda K Fix C Rojas MJ Yasuda T. Tumor necrosis factor α: activity dependent expression and promotion of cortical column sleep in rats. Neuroscience. (2008) 156:71–80. 10.1016/j.neuroscience.2008.06.066 18694809 PMC2654198

[90] Takemiya T Fumizawa K Yamagata K Iwakura Y Kawakami M. Brain interleukin-1 facilitates learning of a water maze spatial memory task in young mice. Front Behav Neurosci. (2017) 11:202. 10.3389/fnbeh.2017.00202 29123474 PMC5662897

[91] Vorhees CV Williams MT. Morris water maze: procedures for assessing spatial and related forms of learning and memory. Nat Protoc. (2006) 1:848–58. 10.1038/nprot.2006.116 17406317 PMC2895266

[92] Song C Phillips AG Leonard BE Horrobin DF. Ethyl-eicosapentaenoic acid ingestion prevents corticosterone-mediated memory impairment induced by central administration of interleukin-1beta in rats. Mol Psychiatry. (2004) 9:630–8. 10.1038/sj.mp.4001462 14699427

[93] Matsumoto Y Yamaguchi T Watanabe S Yamamoto T. Involvement of arachidonic acid cascade in working memory impairment induced by interleukin-1 beta. Neuropharmacology. (2004) 46:1195–200. 10.1016/j.neuropharm.2004.02.012 15111026

[94] Pugh CR Johnson JD Martin D Rudy JW Maier SF Watkins LR. Human immunodeficiency virus-1 coat protein gp120 impairs contextual fear conditioning: a potential role in AIDS related learning and memory impairments. Brain Res. (2000) 861:8–15. 10.1016/S0006-8993(99)02445-2 10751560

[95] Rohleder N Aringer M Boentert M. Role of interleukin-6 in stress, sleep, and fatigue. Ann NY Acad Sci. (2012) 1261:88–96. 10.1111/j.1749-6632.2012.06634.x 22823398

[96] Dantzer R. Neuroimmune interactions: from the brain to the immune system and vice versa. Physiol Rev. (2018) 98:477–504. 10.1152/physrev.00039.2016 29351513 PMC5866360

[97] Terrando N Rei Fidalgo A Vizcaychipi M Cibelli M Ma D Monaco C. The impact of IL-1 modulation on the development of lipopolysaccharide-induced cognitive dysfunction. Crit Care. (2010) 14:R88. 10.1186/cc9019 20470406 PMC2911722

[98] Yirmiya R Winocur G Goshen I. Brain interleukin-1 is involved in spatial memory and passive avoidance conditioning. Neurobiol Learn Mem. (2002) 78:379–89. 10.1006/nlme.2002.4072 12431424

[99] Avital A Goshen I Kamsler A Segal M Iverfeldt K Richter-Levin G. Impaired interleukin-1 signaling is associated with deficits in hippocampal memory processes and neural plasticity. Hippocampus. (2003) 13:826–34. 10.1002/hipo.10135 14620878

[100] Murray CL Obiang P Bannerman D Cunningham C. Endogenous IL-1 in cognitive function and anxiety: a study in IL-1RI^−/−^ mice. PLoS ONE. (2013) 8:e78385. 10.1371/journal.pone.0078385 24205219 PMC3813582

[101] Rossi S Sacchetti L Napolitano F De Chiara V Motta C Studer V. Interleukin-1β causes anxiety by interacting with the endocannabinoid system. J Neurosci. (2012) 32:13896–905. 10.1523/JNEUROSCI.1515-12.2012 23035099 PMC6704788

[102] Dunn AJ Swiergiel AH. Effects of interleukin-1 and endotoxin in the forced swim and tail suspension tests in mice. Pharmacol Biochem Behav. (2005) 81:688–93. 10.1016/j.pbb.2005.04.019 15982728 PMC1975689

[103] Park H-J Shim H-S An K Starkweather A Kim KS Shim I. IL-4 inhibits IL-1β-induced depressive-like behavior and central neurotransmitter alterations. Mediators Inflamm. (2015) 2015:941413. 10.1155/2015/941413 26417153 PMC4568381

[104] Larson SJ. Behavioral and motivational effects of immune-system activation. J Gen Psychol. (2002) 129:401–14. 10.1080/00221300209602104 12494991

[105] Nunes EJ Randall PA Estrada A Epling B Hart EE Lee CA. Effort-related motivational effects of the pro-inflammatory cytokine interleukin 1-beta: studies with the concurrent fixed ratio 5/chow feeding choice task. Psychopharmacology. (2014) 231:727–36. 10.1007/s00213-013-3285-4 24136220 PMC4468782

[106] Yohn SE Arif Y Haley A Tripodi G Baqi Y Müller CE. Effort-related motivational effects of the pro-inflammatory cytokine interleukin-6: pharmacological and neurochemical characterization. Psychopharmacology. (2016) 233:3575–86. 10.1007/s00213-016-4392-9 27497935

[107] Vichaya EG Dantzer R. Inflammation-induced motivational changes: perspective gained by evaluating positive and negative valence systems. Curr Opin Behav Sci. (2018) 22:90–5. 10.1016/j.cobeha.2018.01.008 29888301 PMC5987547

[108] Vichaya EG Hunt SC Dantzer R. Lipopolysaccharide reduces incentive motivation while boosting preference for high reward in mice. Neuropsychopharmacology. (2014) 39:2884–90. 10.1038/npp.2014.141 24917202 PMC4200499

[109] Yang Q Liu R Yu Q Bi Y Liu G. Metabolic regulation of inflammasomes in inflammation. Immunology. (2019) 157: 95–109. 10.1111/imm.13056 30851192 PMC6526672

[110] Belarbi K Cuvelier E Destée A Gressier B Chartier-Harlin M-C. NADPH oxidases in Parkinson's disease: a systematic review. Mol Neurodegener. (2017) 12:84. 10.1186/s13024-017-0225-5 29132391 PMC5683583

[111] Elliott EI Sutterwala FS. Initiation and perpetuation of NLRP3 inflammasome activation and assembly. Immunol Rev. (2015) 265:35–52. 10.1111/imr.12286 25879282 PMC4400874

[112] Liu T Zhang L Joo D Sun S-C. NF-κB signaling in inflammation. Signal Transduct Target Ther. (2017) 2:17023. 10.1038/sigtrans.2017.23 29158945 PMC5661633

[113] Barker BR Taxman DJ Ting JP-Y. Cross-regulation between the IL-1β/IL-18 processing inflammasome and other inflammatory cytokines. Curr Opin Immunol. (2011) 23:591–7. 10.1016/j.coi.2011.07.005 21839623 PMC3380339

[114] Groslambert M Py BF. Spotlight on the NLRP3 inflammasome pathway. J Inflamm Res. (2018) 11:359–74. 10.2147/JIR.S141220 30288079 PMC6161739

[115] Harijith A Ebenezer DL Natarajan V. Reactive oxygen species at the crossroads of inflammasome and inflammation. Front Physiol. (2014) 5:352. 10.3389/fphys.2014.00352 25324778 PMC4179323

[116] Zielinski MR Gerashchenko D Karpova SA Konanki V McCarley RW Sutterwala FS. The NLRP3 inflammasome modulates sleep and NREM sleep delta power induced by spontaneous wakefulness, sleep deprivation and lipopolysaccharide. Brain Behav Immun. (2017) 62:137–50. 10.1016/j.bbi.2017.01.012 28109896 PMC5373953

[117] Dempsey C Rubio Araiz A Bryson KJ Finucane O Larkin C Mills EL. Inhibiting the NLRP3 inflammasome with MCC950 promotes non-phlogistic clearance of amyloid-β and cognitive function in APP/PS1 mice. Brain Behav Immun. (2017) 61:306–16. 10.1016/j.bbi.2016.12.014 28003153

[118] Lei Y Chen C-J Yan X-X Li Z Deng X-H. Early-life lipopolysaccharide exposure potentiates forebrain expression of NLRP3 inflammasome proteins and anxiety-like behavior in adolescent rats. Brain Res. (2017) 1671:43–54. 10.1016/j.brainres.2017.06.014 28655515

[119] Wu P-J Liu H-Y Huang T-N Hsueh Y-P. AIM 2 inflammasomes regulate neuronal morphology and influence anxiety and memory in mice. Sci Rep. (2016) 6:32405. 10.1038/srep32405 27561456 PMC5000013

[120] Wong M-L Inserra A Lewis MD Mastronardi CA Leong L Choo J. Inflammasome signaling affects anxiety- and depressive-like behavior and gut microbiome composition. Mol Psychiatry. (2016) 21:797–805. 10.1038/mp.2016.46 27090302 PMC4879188

[121] Yi Y-S. Role of inflammasomes in inflammatory autoimmune rheumatic diseases. Korean J Physiol Pharmacol. (2018) 22:1–15. 10.4196/kjpp.2018.22.1.1 29302207 PMC5746506

[122] Opipari A Franchi L. Role of inflammasomes in intestinal inflammation and Crohn's disease. Inflamm Bowel Dis. (2015) 21:173–81. 10.1097/MIB.0000000000000230 25517598

[123] Zhang Z Ma X Xia Z Chen J Liu Y Chen Y. NLRP3 inflammasome activation mediates fatigue-like behaviors in mice via neuroinflammation. Neuroscience. (2017) 358:115–23. 10.1016/j.neuroscience.2017.06.048 28684277

[124] McGeough MD Wree A Inzaugarat ME Haimovich A Johnson CD Peña CA. TNF regulates transcription of NLRP3 inflammasome components and inflammatory molecules in cryopyrinopathies. J Clin Invest. (2017) 127:4488–97. 10.1172/JCI90699 29130929 PMC5707143

[125] Kopitar-Jerala N. The role of interferons in inflammation and inflammasome activation. Front Immunol. (2017) 8:873. 10.3389/fimmu.2017.00873 28791024 PMC5525294

[126] Labzin LI Lauterbach MAR Latz E. Interferons and inflammasomes: cooperation and counterregulation in disease. J Allergy Clin Immunol. (2016) 138:37–46. 10.1016/j.jaci.2016.05.010 27373324

[127] Li X Zhang X Pan Y Shi G Ren J Fan H. mTOR regulates NLRP3 inflammasome activation via reactive oxygen species in murine lupus. Acta Biochim Biophys Sin. (2018) 50:888–96. 10.1093/abbs/gmy088 30060081

[128] Cade BE Chen H Stilp AM Louie T Ancoli-Israel S Arens R. Associations of variants In the hexokinase 1 and interleukin 18 receptor regions with oxyhemoglobin saturation during sleep. PLoS Genet. (2019) 15:e1007739. 10.1371/journal.pgen.1007739 30990817 PMC6467367

[129] Moon J-S Hisata S Park M-A DeNicola GM Ryter SW Nakahira K. mTORC1-induced HK1-dependent glycolysis regulates NLRP3 inflammasome activation. Cell Rep. (2015) 12:102–15. 10.1016/j.celrep.2015.05.046 26119735 PMC4858438

[130] Monteiro S Roque S Marques F Correia-Neves M Cerqueira JJ. Brain interference: revisiting the role of IFNγ in the central nervous system. Prog Neurobiol. (2017) 156:149–63. 10.1016/j.pneurobio.2017.05.003 28528956

[131] Masters SL Mielke LA Cornish AL Sutton CE O'Donnell J Cengia LH Roberts AW. Regulation of interleukin-1beta by interferon-gamma is species specific, limited by suppressor of cytokine signalling 1 and influences interleukin-17 production. EMBO Rep. (2010) 11:640–6. 10.1038/embor.2010.93 20596075 PMC2920446

[132] Griffiths HR Gao D Pararasa C. Redox regulation in metabolic programming and inflammation. Redox Biol. (2017) 12:50–7. 10.1016/j.redox.2017.01.023 28212523 PMC5312548

[133] De Nardo D Latz E. NLRP3 inflammasomes link inflammation and metabolic disease. Trends Immunol. (2011) 32:373–9. 10.1016/j.it.2011.05.004 21733753 PMC3151541

[134] Wen H Ting JP-Y O'Neill LAJ. A role for the NLRP3 inflammasome in metabolic diseases–did Warburg miss inflammation? Nat Immunol. (2012) 13:352–7. 10.1038/ni.2228 22430788 PMC4090390

[135] Petit J-M Burlet-Godinot S Magistretti PJ Allaman I. Glycogen metabolism and the homeostatic regulation of sleep. Metab Brain Dis. (2015) 30:263–79. 10.1007/s11011-014-9629-x 25399336 PMC4544655

[136] He F Li J Liu Z Chuang C-C Yang W Zuo L. Redox mechanism of reactive oxygen species in exercise. Front Physiol. (2016) 7:486. 10.3389/fphys.2016.00486 27872595 PMC5097959

[137] Owen L Sunram-Lea SI. Metabolic agents that enhance ATP can improve cognitive functioning: a review of the evidence for glucose, oxygen, pyruvate, creatine, and L-carnitine. Nutrients. (2011) 3:735–55. 10.3390/nu3080735 22254121 PMC3257700

[138] Kennedy C. ATP as a cotransmitter in the autonomic nervous system. Auton Neurosci. (2015) 191:2–15. 10.1016/j.autneu.2015.04.004 26054908

[139] Burnstock G. Purinergic signalling: from discovery to current developments. Exp Physiol. (2014) 99:16–34. 10.1113/expphysiol.2013.071951 24078669 PMC4208685

[140] Gombault A Baron L Couillin I. ATP release and purinergic signaling in NLRP3 inflammasome activation. Front Immunol. (2012) 3:414. 10.3389/fimmu.2012.00414 23316199 PMC3539150

[141] Allard B Longhi MS Robson SC Stagg J. The ectonucleotidases CD39 and CD73: novel checkpoint inhibitor targets. Immunol Rev. (2017) 276:121–44. 10.1111/imr.12528 28258700 PMC5338647

[142] Zielinski MR Taishi P Clinton JM Krueger JM. 5'-Ectonucleotidase-knockout mice lack non-REM sleep responses to sleep deprivation. Eur J Neurosci. (2012) 35:1789–98. 10.1111/j.1460-9568.2012.08112.x 22540145 PMC3370120

[143] Urry E Landolt H-P. Adenosine, caffeine, and performance: from cognitive neuroscience of sleep to sleep pharmacogenetics. Curr Top Behav Neurosci. (2015) 25:331–66. 10.1007/7854_2014_274 24549722

[144] Brown RE Basheer R McKenna JT Strecker RE McCarley RW. Control of sleep and wakefulness. Physiol Rev. (2012) 92:1087–187. 10.1152/physrev.00032.2011 22811426 PMC3621793

[145] Snel J Lorist MM. Effects of caffeine on sleep and cognition. Prog Brain Res. (2011) 190:105–17. 10.1016/B978-0-444-53817-8.00006-2 21531247

[146] Zhang J Wang X Vikash V Ye Q Wu D Liu Y. ROS and ROS-mediated cellular signaling. Oxid Med Cell Longev. (2016) 2016:4350965. 10.1155/2016/4350965 26998193 PMC4779832

[147] Di Dalmazi G Hirshberg J Lyle D Freij JB Caturegli P. Reactive oxygen species in organ-specific autoimmunity. Auto Immun Highlights. (2016) 7:11. 10.1007/s13317-016-0083-0 27491295 PMC4974204

[148] Murphy MP. How mitochondria produce reactive oxygen species. Biochem J. (2009) 417:1–13. 10.1042/BJ20081386 19061483 PMC2605959

[149] Morgan MJ Liu Z. Crosstalk of reactive oxygen species and NF-κB signaling. Cell Res. (2011) 21:103–15. 10.1038/cr.2010.178 21187859 PMC3193400

[150] Zheng X Boyer L Jin M Mertens J Kim Y Ma L. Metabolic reprogramming during neuronal differentiation from aerobic glycolysis to neuronal oxidative phosphorylation. Elife. (2016) 5:e13374. 10.7554/eLife.13374 27282387 PMC4963198

[151] Jha MK Morrison BM. Glia-neuron energy metabolism in health and diseases: new insights into the role of nervous system metabolic transporters. Exp Neurol. (2018) 309:23–31. 10.1016/j.expneurol.2018.07.009 30044944 PMC6156776

[152] Magistretti PJ Allaman I. A cellular perspective on brain energy metabolism and functional imaging. Neuron. (2015) 86:883–901. 10.1016/j.neuron.2015.03.035 25996133

[153] Stincone A Prigione A Cramer T Wamelink MM Campbell K Cheung E. The return of metabolism: biochemistry and physiology of the pentose phosphate pathway. Biol Rev Camb Philos Soc. (2015) 90:927–63. 10.1111/brv.12140 25243985 PMC4470864

[154] Lukyanova LD Kirova YI. Mitochondria-controlled signaling mechanisms of brain protection in hypoxia. Front Neurosci. (2015) 9:320. 10.3389/fnins.2015.00320 26483619 PMC4589588

[155] Gouveia A Bajwa E Klegeris A. Extracellular cytochrome c as an intercellular signaling molecule regulating microglial functions. Biochim Biophys Acta Gen Subj. (2017) 1861:2274–81. 10.1016/j.bbagen.2017.06.017 28652078

[156] Eleftheriadis T Pissas G Liakopoulos V Stefanidis I. Cytochrome C as a potentially clinical useful marker of mitochondrial and cellular damage. Front Immunol. (2016) 7:279. 10.3389/fimmu.2016.00279 27489552 PMC4951490

[157] Ma MW Wang J Dhandapani KM Brann DW. NADPH oxidase 2 regulates NLRP3 inflammasome activation in the brain after traumatic brain injury. Oxid Med Cell Longev. (2017) 2017:6057609. 10.1155/2017/6057609 28785377 PMC5529650

[158] Dolunay A Senol SP Temiz-Resitoglu M Guden DS Sari AN Sahan-Firat S. Inhibition of NLRP3 inflammasome prevents LPS-induced inflammatory hyperalgesia in mice: contribution of NF-κB, Caspase-1/11, ASC, NOX, and NOS isoforms. Inflammation. (2017) 40:366–86. 10.1007/s10753-016-0483-3 27924425

[159] Zhou R Yazdi AS Menu P Tschopp J. A role for mitochondria in NLRP3 inflammasome activation. Nature. (2011) 469:221–5. 10.1038/nature09663 21124315

[160] Munoz FM Gao R Tian Y Henstenburg BA Barrett JE Hu H. Neuronal P2X7 receptor-induced reactive oxygen species production contributes to nociceptive behavior in mice. Sci Rep. (2017) 7:3539. 10.1038/s41598-017-03813-7 28615626 PMC5471238

[161] Boyapati RK Tamborska A Dorward DA Ho G-T. Advances in the understanding of mitochondrial DNA as a pathogenic factor in inflammatory diseases. F1000Research. (2017) 6:169. 10.12688/f1000research.10397.1 28299196 PMC5321122

[162] West AP Khoury-Hanold W Staron M Tal MC Pineda CM Lang SM. Mitochondrial DNA stress primes the antiviral innate immune response. Nature. (2015) 520:553–7. 10.1038/nature14156 25642965 PMC4409480

[163] Kang I Chu CT Kaufman BA. The mitochondrial transcription factor TFAM in neurodegeneration: emerging evidence and mechanisms. FEBS Lett. (2018) 592:793–811. 10.1002/1873-3468.12989 29364506 PMC5851836

[164] Li T Chen ZJ. The cGAS-cGAMP-STING pathway connects DNA damage to inflammation, senescence, and cancer. J Exp Med. (2018) 215:1287–99. 10.1084/jem.20180139 29622565 PMC5940270

[165] Guinn Z Lampe AT Brown DM Petro TM. Significant role for IRF3 in both T cell and APC effector functions during T cell responses. Cell Immunol. (2016) 310:141–9. 10.1016/j.cellimm.2016.08.015 27641636 PMC5125847

[166] Mahmoudian E Khalilnezhad A Gharagozli K Amani D. Thioredoxin-1, redox factor-1 and thioredoxin-interacting protein, mRNAs are differentially expressed in Multiple Sclerosis patients exposed and non-exposed to interferon and immunosuppressive treatments. Gene. (2017) 634:29–36. 10.1016/j.gene.2017.08.021 28844667

[167] Perez-Alvarez MJ Villa Gonzalez M Benito-Cuesta I Wandosell FG. Role of mTORC1 controlling proteostasis after brain ischemia. Front Neurosci. (2018) 12:60. 10.3389/fnins.2018.00060 29497356 PMC5818460

[168] Arbore G Kemper C. A novel “complement-metabolism-inflammasome axis” as a key regulator of immune cell effector function. Eur J Immunol. (2016) 46:1563–73. 10.1002/eji.201546131 27184294 PMC5025719

[169] Perl A. Activation of mTOR (mechanistic target of rapamycin) in rheumatic diseases. Nat Rev Rheumatol. (2016) 12:169–82. 10.1038/nrrheum.2015.172 26698023 PMC5314913

[170] Lai Z-W Hanczko R Bonilla E Caza TN Clair B Bartos A. N-acetylcysteine reduces disease activity by blocking mammalian target of rapamycin in T cells from systemic lupus erythematosus patients: a randomized, double-blind, placebo-controlled trial. Arthritis Rheum. (2012) 64:2937–46. 10.1002/art.34502 22549432 PMC3411859

[171] Saper CB Romanovsky AA Scammell TE. Neural circuitry engaged by prostaglandins during the sickness syndrome. Nat Neurosci. (2012) 15:1088–95. 10.1038/nn.3159 22837039 PMC3748818

[172] Tsuge K Inazumi T Shimamoto A Sugimoto Y. Molecular mechanisms underlying prostaglandin E2-exacerbated inflammation and immune diseases. Int Immunol. (2019) dxz021. [Epub ahead of print]. 10.1093/intimm/dxz021. 30926983

[173] Shaver JL Iacovides S. Sleep in women with chronic pain and autoimmune conditions: a narrative review. Sleep Med Clin. (2018) 13:375–94. 10.1016/j.jsmc.2018.04.008 30098754

[174] Hsiao YH Chen YTYM Tseng CM Wu LA Lin WC Su VY. Sleep disorders and increased risk of autoimmune diseases in individuals without sleep apnea. Sleep. (2015) 38:581–6. 10.5665/sleep.4574 25669189 PMC4355897

[175] Mirrakhimov AE. Obstructive sleep apnea and autoimmune rheumatic disease: is there any link? Inflamm Allergy Drug Targets. (2013) 12:362–7. 10.2174/18715281113129990051 23859748

[176] Kok VC Horng JT Hung GD Xu JL Hung TW Chen YC. Risk of autoimmune disease in adults with chronic insomnia requiring sleep-inducing pills: a population-based longitudinal study. J Gen Intern Med. (2016) 31:1019–26. 10.1007/s11606-016-3717-z 27130621 PMC4978676

[177] Palma BD Gabriel A Colugnati FAB Tufik S. Effects of sleep deprivation on the development of autoimmune disease in an experimental model of systemic lupus erythematosus. Am J Physiol Regul Integr Comp Physiol. (2006) 291:R1527–32. 10.1152/ajpregu.00186.2006 16809486

[178] Young KA Munroe ME Harley JB Guthridge JM Kamen DL Gilkensen GS. Less than 7 hours of sleep per night is associated with transitioning to systemic lupus erythematosus. Lupus. (2018) 27:1524–31. 10.1177/0961203318778368 29804502 PMC6026567

[179] Mahoney CE Cogswell A Koralnik IJ Scammell TE. The neurobiological basis of narcolepsy. Nat Rev Neurosci. (2019) 20:83–93. 10.1038/s41583-018-0097-x 30546103 PMC6492289

[180] Blattner MS de Bruin GS Bucelli RC Day GS. Sleep disturbances are common in patients with autoimmune encephalitis. J Neurol. (2019) 266:1007–15. 10.1007/s00415-019-09230-2 30741377 PMC6421090

[181] Grabovac I Haider S Berner C Lamprecht T Fenzl KH Erlacher L. Sleep quality in patients with rheumatoid arthritis and associations with pain, disability, disease duration, and activity. J Clin Med. (2018) 7:336. 10.3390/jcm7100336 30304765 PMC6210607

[182] Lamis DA Hirsch JK Pugh KC Topciu R Nsamenang SA Goodman A. Perceived cognitive deficits and depressive symptoms in patients with multiple sclerosis: perceived stress and sleep quality as mediators. Mult Scler Relat Disord. (2018) 25:150–5. 10.1016/j.msard.2018.07.019 30081314

[183] Benn BS Lehman Z Kidd SA Miaskowski C Sunwoo BY Ho M. Sleep disturbance and symptom burden in sarcoidosis. Respir Med. (2018) 144S:S35–40. 10.1016/j.rmed.2018.03.021 29628134

[184] Griggs S Redeker NS Grey M. Sleep characteristics in young adults with type 1 diabetes. Diabetes Res Clin Pract. (2019) 150:17–26. 10.1016/j.diabres.2019.02.012 30790611 PMC6525057

[185] van Langenberg DR Yelland GW Robinson SR Gibson PR. Cognitive impairment in Crohn's disease is associated with systemic inflammation, symptom burden and sleep disturbance. United Eur Gastroenterol J. (2017) 5:579–87. 10.1177/2050640616663397 28588890 PMC5446137

[186] Palma BD Tufik S. Increased disease activity is associated with altered sleep architecture in an experimental model of systemic lupus erythematosus. Sleep. (2010) 33:1244–8. 10.1093/sleep/33.9.1244 20857872 PMC2938866

[187] Shen TC Hang LW Liang SJ Huang CC Lin CL Tu CY. Risk of obstructive sleep apnoea in patients with rheumatoid arthritis: a nationwide population-based retrospective cohort study. BMJ Open. (2016) 6:e013151. 10.1136/bmjopen-2016-013151 27895064 PMC5168499

[188] Kang J-H Lin H-C. Obstructive sleep apnea and the risk of autoimmune diseases: a longitudinal population-based study. Sleep Med. (2012) 13:583–8. 10.1016/j.sleep.2012.03.002 22521311

[189] Marrie RA Reider N Cohen J Trojano M Sorensen PS Cutter G. A systematic review of the incidence and prevalence of sleep disorders and seizure disorders in multiple sclerosis. Mult Scler. (2015) 21:342–9. 10.1177/1352458514564486 25533301 PMC4429167

[190] Schell C Schleich R Walker F Yazdi AS Lerche H Röcken M. Restless legs syndrome in psoriasis: an unexpected comorbidity. Eur J Dermatol. (2015) 25:255–60. 10.1684/ejd.2015.252 25786537

[191] Gjevre JA Taylor Gjevre RM. Restless legs syndrome as a comorbidity in rheumatoid arthritis. Autoimmune Dis. (2013) 2013:352782. 10.1155/2013/352782 23840943 PMC3694367

[192] Ning P Hu F Yang B Shen Q Zhao Q Huang H. Systematic review and meta-analysis of observational studies to understand the prevalence of restless legs syndrome in multiple sclerosis: an update. Sleep Med. (2018) 50:97–104. 10.1016/j.sleep.2018.05.039 30025277

[193] Jackson ML Bruck D. Sleep abnormalities in chronic fatigue syndrome/myalgic encephalomyelitis: a review. J Clin Sleep Med. (2012) 8:719–28. 10.5664/jcsm.2276 23243408 PMC3501671

[194] Mifflin KA Kerr BJ. Pain in autoimmune disorders. J Neurosci Res. (2017) 95:1282–94. 10.1002/jnr.23844 27448322

[195] McBeth J Wilkie R Bedson J Chew-Graham C Lacey RJ. Sleep disturbance and chronic widespread pain. Curr Rheumatol Rep. (2015) 17:469. 10.1007/s11926-014-0469-9 25604572

[196] Slater G Steier J. Excessive daytime sleepiness in sleep disorders. J Thorac Dis. (2012) 4:608–16. 10.3978/j.issn.2072-1439.2012.10.07 23205286 PMC3506799

[197] Mullins HM Cortina JM Drake CL Dalal RS. Sleepiness at work: a review and framework of how the physiology of sleepiness impacts the workplace. J Appl Psychol. (2014) 99:1096–12. 10.1037/a0037885 25384205

[198] Cellini N. Memory consolidation in sleep disorders. Sleep Med Rev. (2017) 35:101–12. 10.1016/j.smrv.2016.09.003 27765468

[199] Batool-Anwar S Kales SN Patel SR Varvarigou V DeYoung PN Malhotra A. Obstructive sleep apnea and psychomotor vigilance task performance. Nat Sci Sleep. (2014) 6:65–71. 10.2147/NSS.S53721 24920941 PMC4043718

[200] Altena E Van Der Werf YD Strijers RLM Van Someren EJW. Sleep loss affects vigilance: effects of chronic insomnia and sleep therapy. J Sleep Res. (2008) 17:335–43. 10.1111/j.1365-2869.2008.00671.x 18844819

[201] Spiegelhalder K Regen W Nanovska S Baglioni C Riemann D. Comorbid sleep disorders in neuropsychiatric disorders across the life cycle. Curr Psychiatry Rep. (2013) 15:364. 10.1007/s11920-013-0364-5 23636987

[202] Gaspar LS Álvaro AR Moita J Cavadas C. Obstructive sleep apnea and hallmarks of aging. Trends Mol Med. (2017) 23:675–92. 10.1016/j.molmed.2017.06.006 28739207

[203] Ranjbaran Z Keefer L Stepanski E Farhadi A Keshavarzian A. The relevance of sleep abnormalities to chronic inflammatory conditions. Inflamm Res. (2007) 56:51–7. 10.1007/s00011-006-6067-1 17431741

[204] Van Dongen HPA Maislin G Mullington JM Dinges DF. The cumulative cost of additional wakefulness: dose-response effects on neurobehavioral functions and sleep physiology from chronic sleep restriction and total sleep deprivation. Sleep. (2003) 26:117–26. 10.1093/sleep/26.2.117 12683469

[205] Goel N Basner M Rao H Dinges DF. Circadian rhythms, sleep deprivation, and human performance. Prog Mol Biol Transl Sci. (2013) 119:155–90. 10.1016/B978-0-12-396971-2.00007-5 23899598 PMC3963479

[206] Mantua J Spencer RMC Exploring the nap paradox: are mid-day sleep bouts a friend or foe? Sleep Med. (2017) 37:88–97. 10.1016/j.sleep.2017.01.019 28899546 PMC5598771

[207] Powell DJH Liossi C Schlotz W Moss-Morris R. Tracking daily fatigue fluctuations in multiple sclerosis: ecological momentary assessment provides unique insights. J Behav Med. (2017) 40:772–83. 10.1007/s10865-017-9840-4 28281106 PMC5613039

[208] Unnikrishnan D Jun J Polotsky V. Inflammation in sleep apnea: an update. Rev Endocr Metab Disord. (2015) 16:25–34. 10.1007/s11154-014-9304-x 25502450 PMC4346503

[209] Mondello S Kobeissy F Mechref Y Zhao J Talih FR Cosentino F. Novel biomarker signatures for idiopathic REM sleep behavior disorder: a proteomic and system biology approach. Neurology. (2018) 91:e1710–5. 10.1212/WNL.0000000000006439 30258025

[210] Pejovic S Vgontzas AN Neurobiological Disturbances in Insomnia: Clinical Utility of Objective Measures of Sleep. Insomnia: Diagnosis and Treatment. London: CRC Press (2016). p. 65–76.

[211] Pals DT Lawson JA Couch SJ. Rat model for evaluating inhibitors of human renin. J Pharmacol Methods. (1990) 23:239–45. 10.1016/0160-5402(90)90052-M 2196401

[212] Gerashchenko D Schmidt MA Zielinski MR Moore ME Wisor JP. Sleep state dependence of optogenetically evoked responses in neuronal nitric oxide synthase-positive cells of the cerebral cortex. Neuroscience. (2018) 379:189–201. 10.1016/j.neuroscience.2018.02.006 29438803 PMC6311348

[213] Imeri L Bianchi S Opp MR. Inhibition of caspase-1 in rat brain reduces spontaneous nonrapid eye movement sleep and nonrapid eye movement sleep enhancement induced by lipopolysaccharide. Am J Physiol Regul Integr Comp Physiol. (2006) 291:R197–204. 10.1152/ajpregu.00828.2005 16455762

[214] Xia M Li X Yang L Ren J Sun G Qi S The ameliorative effect of fluoxetine on neuroinflammation induced by sleep deprivation. J Neurochem. (2017) 146:63–75. 10.1111/jnc.14272 29222907

[215] Li X Liang S Li Z Li S Xia M Verkhratsky A. Leptin increases expression of 5-HT2B receptors in astrocytes thus enhancing action of fluoxetine on the depressive behavior induced by sleep deprivation. Front psychiatry. (2018) 9:734. 10.3389/fpsyt.2018.00734 30666218 PMC6330762

[216] Yoon DW Shin S Choi J Shin C Lee C Lee J Sleep fragmentation induces activation of NOD-like receptor protein-3 inflammasome in rat hippocampus. Sleep Med Res. (2017) 8:26–32. 10.17241/smr.2017.00017

[217] Menet JS Pescatore S Rosbash M. CLOCK:BMAL1 is a pioneer-like transcription factor. Genes Dev. (2014) 28:8–13. 10.1101/gad.228536.113 24395244 PMC3894415

[218] Dubowy C Sehgal A. Circadian rhythms and sleep in *Drosophila melanogaster*. Genetics. (2017) 205:1373–97. 10.1534/genetics.115.185157 28360128 PMC5378101

[219] Lee B Li A Hansen KF Cao R Yoon JH Obrietan K. CREB influences timing and entrainment of the SCN circadian clock. J Biol Rhythms. (2010) 25:410–20. 10.1177/0748730410381229 21135157 PMC3529591

[220] Hardin PE Yu W. Circadian transcription: passing the HAT to CLOCK. Cell. (2006) 125:424–6. 10.1016/j.cell.2006.04.010 16678086

[221] Rivers-Auty J Daniels MJD Colliver I Robertson DL Brough D. Redefining the ancestral origins of the interleukin-1 superfamily. Nat Commun. (2018) 9:1156. 10.1038/s41467-018-03362-1 29559685 PMC5861070

[222] Roerink ME van der Schaaf ME Dinarello CA Knoop H van der Meer JWM. Interleukin-1 as a mediator of fatigue in disease: a narrative review. J Neuroinflammation. (2017) 14:16. 10.1186/s12974-017-0796-7 28109186 PMC5251329

[223] Lagishetty V Parthasarathy PT Phillips O Fukumoto J Cho Y Fukumoto I. Dysregulation of CLOCK gene expression in hyperoxia-induced lung injury. Am J Physiol Cell Physiol. (2014) 306:C999–1007. 10.1152/ajpcell.00064.2013 24696144 PMC4042094

[224] Sutton CE Finlay CM Raverdeau M Early JO DeCourcey J Zaslona Z. Loss of the molecular clock in myeloid cells exacerbates T cell-mediated CNS autoimmune disease. Nat Commun. (2017) 8:1923. 10.1038/s41467-017-02111-0 29234010 PMC5727202

[225] Lin G-J Huang S-H Chen S-J Wang C-H Chang D-M Sytwu H-K. Modulation by melatonin of the pathogenesis of inflammatory autoimmune diseases. Int J Mol Sci. (2013) 14:11742–66. 10.3390/ijms140611742 23727938 PMC3709754

[226] Matura LA Malone S Jaime-Lara R Riegel B. A systematic review of biological mechanisms of fatigue in chronic illness. Biol Res Nurs. (2018) 20:410–21. 10.1177/1099800418764326 29540066 PMC6346311

[227] Silverman MN Heim CM Nater UM Marques AH Sternberg EM. Neuroendocrine and immune contributors to fatigue. PM R. (2010) 2:338–46. 10.1016/j.pmrj.2010.04.008 20656615 PMC2933136

[228] Karagkouni A Alevizos M Theoharides TC. Effect of stress on brain inflammation and multiple sclerosis. Autoimmun Rev. (2013) 12:947–53. 10.1016/j.autrev.2013.02.006 23537508

[229] Bellavance M-A Rivest S. The HPA - immune axis and the immunomodulatory actions of glucocorticoids in the brain. Front Immunol. (2014) 5:136. 10.3389/fimmu.2014.00136 24744759 PMC3978367

[230] Scanzano A Cosentino M. Adrenergic regulation of innate immunity: a review. Front Pharmacol. (2015) 6:171. 10.3389/fphar.2015.00171 26321956 PMC4534859

[231] Ballanti E Perricone C Greco E Ballanti M Di Muzio G Chimenti MS. Complement and autoimmunity. Immunol Res. (2013) 56:477–91. 10.1007/s12026-013-8422-y 23615835

[232] Barnes MA Carson MJ Nair MG. Non-traditional cytokines: how catecholamines and adipokines influence macrophages in immunity, metabolism and the central nervous system. Cytokine. (2015) 72:210–9. 10.1016/j.cyto.2015.01.008 25703786 PMC4590987

[233] Gyoneva S Traynelis SF. Norepinephrine modulates the motility of resting and activated microglia via different adrenergic receptors. J Biol Chem. (2013) 288:15291–302. 10.1074/jbc.M113.458901 23548902 PMC3663549

[234] Laureys G Gerlo S Spooren A Demol F De Keyser J Aerts JL. β-adrenergic agonists modulate TNF-α induced astrocytic inflammatory gene expression and brain inflammatory cell populations. J Neuroinflammation. (2014) 11:21. 10.1186/1742-2094-11-21 24479486 PMC3942172

[235] Liu Y-Z Wang Y-X Jiang C-L. Inflammation: the common pathway of stress-related diseases. Front Hum Neurosci. (2017) 11:316. 10.3389/fnhum.2017.00316 28676747 PMC5476783

[236] Busillo JM Azzam KM Cidlowski JA. Glucocorticoids sensitize the innate immune system through regulation of the NLRP3 inflammasome. J Biol Chem. (2011) 286:38703–13. 10.1074/jbc.M111.275370 21940629 PMC3207479

[237] Dickmeis T. Glucocorticoids and the circadian clock. J Endocrinol. (2009) 200:3–22. 10.1677/JOE-08-0415 18971218

[238] Leproult R Copinschi G Buxton O Van Cauter E. Sleep loss results in an elevation of cortisol levels the next evening. Sleep. (1997) 20:865–70. 9415946

[239] Miller GE Murphy MLM Cashman R Ma R Ma J Arevalo JM. Greater inflammatory activity and blunted glucocorticoid signaling in monocytes of chronically stressed caregivers. Brain Behav Immun. (2014) 41:191–9. 10.1016/j.bbi.2014.05.016 25242587 PMC4973629

[240] Prough RA Clark BJ Klinge CM. Novel mechanisms for DHEA action. J Mol Endocrinol. (2016) 56:R139–55. 10.1530/JME-16-0013 26908835

[241] Téllez N Comabella M Julià E Río J Tintoré M Brieva L. Fatigue in progressive multiple sclerosis is associated with low levels of dehydroepiandrosterone. Mult Scler. (2006) 12:487–94. 10.1191/135248505ms1322oa 16900763

[242] Sawalha AH Kovats S. Dehydroepiandrosterone in systemic lupus erythematosus. Curr Rheumatol Rep. (2008) 10:286–91. 10.1007/s11926-008-0046-1 18662508 PMC2701249

[243] Harrington ME. Neurobiological studies of fatigue. Prog Neurobiol. (2012) 99:93–105. 10.1016/j.pneurobio.2012.07.004 22841649 PMC3479364

[244] Galtress T Marshall AT Kirkpatrick K. Motivation and timing: clues for modeling the reward system. Behav Process. (2012) 90:142–53. 10.1016/j.beproc.2012.02.014 22421220 PMC3335954

[245] Miller AH Haroon E Raison CL Felger JC. Cytokine targets in the brain: impact on neurotransmitters and neurocircuits. Depress Anxiety. (2013) 30:297–306. 10.1002/da.22084 23468190 PMC4141874

[246] Gottesmann C. GABA mechanisms and sleep. Neuroscience. (2002) 111:231–9. 10.1016/S0306-4522(02)00034-9 11983310

[247] Goddard AW. Cortical and subcortical gamma amino acid butyric acid deficits in anxiety and stress disorders: clinical implications. World J Psychiatry. (2016) 6:43–53. 10.5498/wjp.v6.i1.43 27014597 PMC4804267

[248] Szymusiak R McGinty D. Hypothalamic regulation of sleep and arousal. Ann NY Acad Sci. (2008) 1129:275–86. 10.1196/annals.1417.027 18591488

[249] Patel RR Khom S Steinman MQ Varodayan FP Kiosses WB Hedges DM. IL-1β expression is increased and regulates GABA transmission following chronic ethanol in mouse central amygdala. Brain Behav Immun. (2019) 75:208–19. 10.1016/j.bbi.2018.10.009 30791967 PMC6383367

[250] Cordeiro LMS Rabelo PCR Moraes MM. Physical exercise-induced fatigue: the role of serotonergic and dopaminergic systems. Braz J Med Biol Res. (2017) 50:e6432. 10.1590/1414-431x20176432 29069229 PMC5649871

[251] Liu Y Zhao J Guo W. Emotional roles of mono-aminergic neurotransmitters in major depressive disorder and anxiety disorders. Front Psychol. (2018) 9:2201. 10.3389/fpsyg.2018.02201 30524332 PMC6262356

[252] Holst SC Landolt H-P. Sleep-wake neurochemistry. Sleep Med Clin. (2018) 13:137–46. 10.1016/j.jsmc.2018.03.002 29759265

[253] Quentin E Belmer A Maroteaux L. Somato-dendritic regulation of raphe serotonin neurons; a key to antidepressant action. Front Neurosci. (2018) 12:982. 10.3389/fnins.2018.00982 30618598 PMC6307465

[254] Nero D Bowditch N Pickert S MacIntyre RJ. A genetic and molecular analysis of P-induced mutations at the glucose-6-phosphate dehydrogenase locus in *Drosophila melanogaster*. Mol Gen Genet. (1989) 219:429–38. 10.1007/BF00259616 2560135

[255] Watts SW Morrison SF Davis RP Barman SM. Serotonin and blood pressure regulation. Pharmacol Rev. (2012) 64:359–88. 10.1124/pr.111.004697 22407614 PMC3310484

[256] Shintani F Kanba S Nakaki T Nibuya M Kinoshita N Suzuki E. Interleukin-1 beta augments release of norepinephrine, dopamine, and serotonin in the rat anterior hypothalamus. J Neurosci. (1993) 13:3574–81. 10.1523/JNEUROSCI.13-08-03574.1993 8393485 PMC6576546

[257] Cotecchia S Stanasila L Diviani D. Protein-protein interactions at the adrenergic receptors. Curr Drug Targets. (2012) 13:15–27. 10.2174/138945012798868489 21777184 PMC3290771

[258] McMorris T Barwood M Corbett J. Central fatigue theory and endurance exercise: Toward an interoceptive model. Neurosci Biobehav Rev. (2018) 93:93–107. 10.1016/j.neubiorev.2018.03.024 29608992

[259] Vitrac C Benoit-Marand M. Monoaminergic modulation of motor cortex function. Front Neural Circuits. (2017) 11:72. 10.3389/fncir.2017.00072 29062274 PMC5640772

[260] Dahlstroem A Fuxe K Evidence for the existence of monoamine-containing neurons in the central nervous system. I. Demonstration of monoamines in the cell bodies of brain stem neurons. Acta Physiol Scand Suppl. (1964) 232:1–55. 14229500

[261] Aston-Jones G Bloom FE. Activity of norepinephrine-containing locus coeruleus neurons in behaving rats anticipates fluctuations in the sleep-waking cycle. J Neurosci. (1981) 1:876–86. 10.1523/JNEUROSCI.01-08-00876.1981 7346592 PMC6564235

[262] Rho H-J Kim J-H Lee S-H. Function of selective neuromodulatory projections in the mammalian cerebral cortex: comparison between cholinergic and noradrenergic systems. Front Neural Circuits. (2018) 12:47. 10.3389/fncir.2018.00047 29988373 PMC6023998

[263] Hasbi A O'Dowd BF George SR. Dopamine D1-D2 receptor heteromer signaling pathway in the brain: emerging physiological relevance. Mol Brain. (2011) 4:26. 10.1186/1756-6606-4-26 21663703 PMC3138392

[264] Miller GM. The emerging role of trace amine-associated receptor 1 in the functional regulation of monoamine transporters and dopaminergic activity. J Neurochem. (2011) 116:164–76. 10.1111/j.1471-4159.2010.07109.x 21073468 PMC3005101

[265] Liu C Kaeser PS. Mechanisms and regulation of dopamine release. Curr Opin Neurobiol. (2019) 57:46–53. 10.1016/j.conb.2019.01.001 30769276 PMC6629510

[266] Sadeghniiat-Haghighi K Yazdi Z. Fatigue management in the workplace. Ind Psychiatry J. (2015) 24:12–7. 10.4103/0972-6748.160915 26257477 PMC4525425

[267] Wylie GR Dobryakova E DeLuca J Chiaravalloti N Essad K Genova H. Cognitive fatigue in individuals with traumatic brain injury is associated with caudate activation. Sci Rep. (2017) 7:8973. 10.1038/s41598-017-08846-6 28827779 PMC5567054

[268] Taber KH Black DN Porrino LJ Hurley RA. Neuroanatomy of dopamine: reward and addiction. J Neuropsychiatry Clin Neurosci. (2012) 24:1–4. 10.1176/appi.neuropsych.24.1.1 22450608

[269] Ranaldi R. Dopamine and reward seeking: the role of ventral tegmental area. Rev Neurosci. (2014) 25:621–30. 10.1515/revneuro-2014-0019 24887956

[270] Russo SJ Nestler EJ. The brain reward circuitry in mood disorders. Nat Rev Neurosci. (2013) 14:609–25. 10.1038/nrn3381 23942470 PMC3867253

[271] Ziebell JM Morganti-Kossmann MC. Involvement of pro- and anti-inflammatory cytokines and chemokines in the pathophysiology of traumatic brain injury. Neurotherapeutics. (2010) 7:22–30. 10.1016/j.nurt.2009.10.016 20129494 PMC5084109

[272] Dobryakova E Genova HM DeLuca J Wylie GR. The dopamine imbalance hypothesis of fatigue in multiple sclerosis and other neurological disorders. Front Neurol. (2015) 6:52. 10.3389/fneur.2015.00052 25814977 PMC4357260

[273] Parsons ME Ganellin CR Histamine and its receptors. Br J Pharmacol. (2006) 147SupplS1:S127–35. 10.1038/sj.bjp.0706440 16402096 PMC1760721

[274] Passani MB Ballerini C. Histamine and neuroinflammation: insights from murine experimental autoimmune encephalomyelitis. Front Syst Neurosci. (2012) 6:32. 10.3389/fnsys.2012.00032 22563309 PMC3342557

[275] Shan L Dauvilliers Y Siegel JM. Interactions of the histamine and hypocretin systems in CNS disorders. Nat Rev Neurol. (2015) 11:401–13. 10.1038/nrneurol.2015.99 26100750 PMC8744538

[276] Loy BD O'Connor PJ. The effect of histamine on changes in mental energy and fatigue after a single bout of exercise. Physiol Behav. (2016) 153:7–18. 10.1016/j.physbeh.2015.10.016 26482543

[277] Branco ACCC Yoshikawa FSY Pietrobon AJ Sato MN. Role of histamine in modulating the immune response and inflammation. Mediators Inflamm. (2018) 2018:9524075. 10.1155/2018/9524075 30224900 PMC6129797

[278] Akerman S Williamson DJ Kaube H Goadsby PJ. The role of histamine in dural vessel dilation. Brain Res. (2002) 956:96–102. 10.1016/S0006-8993(02)03485-6 12426051

[279] Scammell TE Arrigoni E Lipton JO. Neural circuitry of wakefulness and sleep. Neuron. (2017) 93:747–65. 10.1016/j.neuron.2017.01.014 28231463 PMC5325713

[280] Panula P Chazot PL Cowart M Gutzmer R Leurs R Liu WL. International union of basic and clinical pharmacology. XCVIII. Histamine receptors. Pharmacol Rev. (2015) 67:601–55. 10.1124/pr.114.010249 26084539 PMC4485016

[281] Thakkar MM. Histamine in the regulation of wakefulness. Sleep Med Rev. (2011) 15:65–74. 10.1016/j.smrv.2010.06.004 20851648 PMC3016451

[282] Church MK Church DS. Pharmacology of antihistamines. Indian J Dermatol. (2013) 58:219–24. 10.4103/0019-5154.110832 23723474 PMC3667286

[283] Musio S Gallo B Scabeni S Lapilla M Poliani PL Matarese G. A key regulatory role for histamine in experimental autoimmune encephalomyelitis: disease exacerbation in histidine decarboxylase-deficient mice. J Immunol. (2006) 176:17–26. 10.4049/jimmunol.176.1.17 16365391

[284] Saligrama N Noubade R Case LK del Rio R Teuscher C. Combinatorial roles for histamine H1-H2 and H3-H4 receptors in autoimmune inflammatory disease of the central nervous system. Eur J Immunol. (2012) 42:1536–46. 10.1002/eji.201141859 22678907 PMC3508704

[285] Walker MC van der Donk WA. The many roles of glutamate in metabolism. J Ind Microbiol Biotechnol. (2016) 43:419–30. 10.1007/s10295-015-1665-y 26323613 PMC4753154

[286] Lau A Tymianski M. Glutamate receptors, neurotoxicity and neurodegeneration. Pflugers Arch. (2010) 460:525–42. 10.1007/s00424-010-0809-1 20229265

[287] Rönnbäck L Hansson E. On the potential role of glutamate transport in mental fatigue. J Neuroinflammation. (2004) 1:22. 10.1186/1742-2094-1-22 15527505 PMC533886

[288] Moussawi K Riegel A Nair S Kalivas PW. Extracellular glutamate: functional compartments operate in different concentration ranges. Front Syst Neurosci. (2011) 5:94. 10.3389/fnsys.2011.00094 22275885 PMC3254064

[289] Langer J Gerkau NJ Derouiche A Kleinhans C Moshrefi-Ravasdjani B Fredrich M. Rapid sodium signaling couples glutamate uptake to breakdown of ATP in perivascular astrocyte endfeet. Glia. (2017) 65:293–308. 10.1002/glia.23092 27785828

[290] Feng LR Fernández-Martínez JL Zaal KJM deAndrés-Galiana EJ Wolff BS Saligan LN. mGluR5 mediates post-radiotherapy fatigue development in cancer patients. Transl Psychiatry. (2018) 8:110. 10.1038/s41398-018-0161-3 29849049 PMC5976668

[291] Saligan LN Farmer C Ballard ED Kadriu B Zarate CA. Disentangling the association of depression on the anti-fatigue effects of ketamine. J Affect Disord. (2019) 244:42–5. 10.1016/j.jad.2018.10.089 30312839 PMC6226316

[292] Gardoni F Boraso M Zianni E Corsini E Galli CL Cattabeni F. Distribution of interleukin-1 receptor complex at the synaptic membrane driven by interleukin-1β and NMDA stimulation. J Neuroinflammation. (2011) 8:14. 10.1186/1742-2094-8-14 21314939 PMC3045339

[293] Pearson VL Rothwell NJ Toulmond S. Excitotoxic brain damage in the rat induces interleukin-1beta protein in microglia and astrocytes: correlation with the progression of cell death. Glia. (1999) 25:311–23. 10.1002/(SICI)1098-1136(19990215)25:4&lt;311::AID-GLIA1&gt;3.3.CO;2-5 10028914

[294] Szutowicz A Bielarczyk H Jankowska-Kulawy A Pawełczyk T Ronowska A. Acetyl-CoA the key factor for survival or death of cholinergic neurons in course of neurodegenerative diseases. Neurochem Res. (2013) 38:1523–42. 10.1007/s11064-013-1060-x 23677775 PMC3691476

[295] Picciotto MR Higley MJ Mineur YS. Acetylcholine as a neuromodulator: cholinergic signaling shapes nervous system function and behavior. Neuron. (2012) 76:116–29. 10.1016/j.neuron.2012.08.036 23040810 PMC3466476

[296] Watson CJ Baghdoyan HA Lydic R. Neuropharmacology of sleep and wakefulness. Sleep Med Clin. (2010) 5:513–28. 10.1016/j.jsmc.2010.08.003 21278831 PMC3026477

[297] Meeusen R Roelands B. Central fatigue and neurotransmitters, can thermoregulation be manipulated? Scand J Med Sci Sports. (2010) 20(Suppl. 3):19–28. 10.1111/j.1600-0838.2010.01205.x 21029187

[298] Jordan B Mehl T Schweden TLK Menge U Zierz S. Assessment of physical fatigability and fatigue perception in myasthenia gravis. Muscle Nerve. (2017) 55:657–63. 10.1002/mus.25386 27543741

[299] Gilhus NE Verschuuren JJ. Myasthenia gravis: subgroup classification and therapeutic strategies. Lancet Neurol. (2015) 14:1023–36. 10.1016/S1474-4422(15)00145-3 26376969

[300] Hsu W-Y Lane H-Y Lin C-H. Medications used for cognitive enhancement in patients with schizophrenia, bipolar disorder, Alzheimer's disease, and Parkinson's disease. Front Psychiatry. (2018) 9:91. 10.3389/fpsyt.2018.00091 29670547 PMC5893641

[301] Shytle RD Mori T Townsend K Vendrame M Sun N Zeng J. Cholinergic modulation of microglial activation by alpha 7 nicotinic receptors. J Neurochem. (2004) 89:337–43. 10.1046/j.1471-4159.2004.02347.x 15056277

[302] Hecker A Küllmar M Wilker S Richter K Zakrzewicz A Atanasova S. Phosphocholine-modified macromolecules and canonical nicotinic agonists inhibit ATP-induced IL-1β release. J Immunol. (2015) 195:2325–34. 10.4049/jimmunol.1400974 26202987

[303] Brambilla D Barajon I Bianchi S Opp MR Imeri L. Interleukin-1 inhibits putative cholinergic neurons *in vitro* and REM sleep when microinjected into the rat laterodorsal tegmental nucleus. Sleep. (2010) 33:919–29. 10.1093/sleep/33.7.919 20614852 PMC2894434

[304] Chaudhuri A Behan PO. Fatigue in neurological disorders. Lancet. (2004) 363:978–88. 10.1016/S0140-6736(04)15794-2 15043967

[305] Christie AD Seery E Kent JA. Physical activity, sleep quality, and self-reported fatigue across the adult lifespan. Exp Gerontol. (2016) 77:7–11. 10.1016/j.exger.2016.02.001 26853493 PMC4808431

[306] Sakurai T. The role of orexin in motivated behaviours. Nat Rev Neurosci. (2014) 15:719–31. 10.1038/nrn3837 25301357

[307] Tsujino N Sakurai T. Orexin/hypocretin: a neuropeptide at the interface of sleep, energy homeostasis, and reward system. Pharmacol Rev. (2009) 61:162–76. 10.1124/pr.109.001321 19549926

[308] Sakurai T. Orexin: a link between energy homeostasis and adaptive behaviour. Curr Opin Clin Nutr Metab Care. (2003) 6:353–60. 10.1097/00075197-200307000-00001 12806206

[309] Ohno K Sakurai T. Orexin neuronal circuitry: role in the regulation of sleep and wakefulness. Front Neuroendocrinol. (2008) 29:70–87. 10.1016/j.yfrne.2007.08.001 17910982

[310] Ryan NP Beauchamp MH Beare R Coleman L Ditchfield M Kean M. Uncovering cortico-striatal correlates of cognitive fatigue in pediatric acquired brain disorder: evidence from traumatic brain injury. Cortex. (2016) 83:222–30. 10.1016/j.cortex.2016.07.020 27603573

[311] Grossberg AJ Zhu X Leinninger GM Levasseur PR Braun TP Myers MG Jr. Inflammation-induced lethargy is mediated by suppression of orexin neuron activity. J Neurosci. (2011) 31:11376–86. 10.1523/JNEUROSCI.2311-11.2011 21813697 PMC3155688

[312] Erickson MA Banks WA. Neuroimmune axes of the blood-brain barriers and blood-brain interfaces: bases for physiological regulation, disease states, and pharmacological interventions. Pharmacol Rev. (2018) 70:278–314. 10.1124/pr.117.014647 29496890 PMC5833009

[313] Bordoni B Purgol S Bizzarri A Modica M Morabito B. The influence of breathing on the central nervous system. Cureus. (2018) 10:e2724. 10.7759/cureus.2724 30083485 PMC6070065

[314] García-Medina NE Miranda MI. Nucleus of the solitary tract chemical stimulation induces extracellular norepinephrine release in the lateral and basolateral amygdala. Brain Stimul. (2013) 6:198–201. 10.1016/j.brs.2012.03.020 22543094

[315] Zeinvand-Lorestani M Kalantari H Khodayar MJ Teimoori A Saki N Ahangarpour A. Autophagy upregulation as a possible mechanism of arsenic induced diabetes. Sci Rep. (2018) 8:11960. 10.1038/s41598-018-30439-0 30097599 PMC6086829

[316] Luo T Yu S Cai S Zhang Y Jiao Y Yu T. Parabrachial neurons promote behavior and electroencephalographic arousal from general anesthesia. Front Mol Neurosci. (2018) 11:420. 10.3389/fnmol.2018.00420 30564094 PMC6288364

[317] Layé S Bluthé RM Kent S Combe C Médina C Parnet P. Subdiaphragmatic vagotomy blocks induction of IL-1 beta mRNA in mice brain in response to peripheral LPS. Am J Physiol. (1995) 268(5 Pt 2):R1327–31. 10.1152/ajpregu.1995.268.5.R1327 7771597

[318] Hansen MK Taishi P Chen Z Krueger JM. Vagotomy blocks the induction of interleukin-1beta (IL-1beta) mRNA in the brain of rats in response to systemic IL-1beta. J Neurosci. (1998) 18:2247–53. 10.1523/JNEUROSCI.18-06-02247.1998 9482809 PMC6792909

[319] Kubota T Fang J Guan Z Brown RA Krueger JM. Vagotomy attenuates tumor necrosis factor-alpha-induced sleep and EEG delta-activity in rats. Am J Physiol Regul Integr Comp Physiol. (2001) 280:R1213–20. 10.1152/ajpregu.2001.280.4.R1213 11247847

[320] Hansen MK Krueger JM. Subdiaphragmatic vagotomy blocks the sleep- and fever-promoting effects of interleukin-1beta. Am J Physiol. (1997) 273(4 Pt 2):R1246–53. 10.1152/ajpregu.1997.273.4.R1246 9362287

[321] Hosoi T Okuma Y Nomura Y. Electrical stimulation of afferent vagus nerve induces IL-1beta expression in the brain and activates HPA axis. Am J Physiol Regul Integr Comp Physiol. (2000) 279:R141–7. 10.1152/ajpregu.2000.279.1.R141 10896875

[322] Kaur C Ling EA. The circumventricular organs. Histol Histopathol. (2017) 32:879–92. 10.14670/HH-11-881 28177105

[323] Wang QP Guan JL Pan W Kastin AJ Shioda S. A diffusion barrier between the area postrema and nucleus tractus solitarius. Neurochem Res. (2008) 33:2035–43. 10.1007/s11064-008-9676-y 18373195

[324] Miyata S. New aspects in fenestrated capillary and tissue dynamics in the sensory circumventricular organs of adult brains. Front Neurosci. (2015) 9:390. 10.3389/fnins.2015.00390 26578857 PMC4621430

[325] Chavan SS Tracey KJ. Essential Neuroscience in Immunology. J Immunol. (2017) 198:3389–97. 10.4049/jimmunol.1601613 28416717 PMC5426063

[326] Corsi-Zuelli FM das G Brognara F Quirino GFDS Hiroki CH Fais RS Del-Ben CM Neuroimmune interactions in schizophrenia: focus on vagus nerve stimulation and activation of the alpha-7 nicotinic acetylcholine receptor. Front Immunol. (2017) 8:618 10.3389/fimmu.2017.00618 28620379 PMC5449450

[327] Koopman FA Chavan SS Miljko S Grazio S Sokolovic S Schuurman PR. Vagus nerve stimulation inhibits cytokine production and attenuates disease severity in rheumatoid arthritis. Proc Natl Acad Sci USA. (2016) 113:8284–9. 10.1073/pnas.1605635113 27382171 PMC4961187

[328] Tarn J Legg S Mitchell S Simon B Ng WF. The effects of noninvasive vagus nerve stimulation on fatigue and immune responses in patients with primary sjögren's syndrome. Neuromodulation. (2019) 22:580–5. 10.1111/ner.12879 30328647

[329] Zhou H Wang Y Bi K Qi H Song S Zhou M. Serum-soluble TRAIL: a potential biomarker for disease activity in myositis patients. Clin Rheumatol. (2019) 38:1425–31. 10.1007/s10067-018-04418-9 30645753

[330] Nakatomi Y Mizuno K Ishii A Wada Y Tanaka M Tazawa S Neuroinflammation in patients with chronic fatigue syndrome/myalgic encephalomyelitis: an ^11^C-(R)-PK11195 PET study. J Nucl Med. (2014) 55:945–50. 10.2967/jnumed.113.131045 24665088

[331] Joris PJ Mensink RP Adam TC Liu TT. Cerebral blood flow measurements in adults: a review on the effects of dietary factors and exercise. Nutrients. (2018) 10:530. 10.3390/nu10050530 29693564 PMC5986410

[332] Sidorenko I Turova V Botkin N Eckardt L Alves-Pinto A Felderhoff-Müser U. Modeling cerebral blood flow dependence on carbon dioxide and mean arterial blood pressure in the immature brain with accounting for the germinal matrix. Front Neurol. (2018) 9:812. 10.3389/fneur.2018.00812 30356709 PMC6189337

[333] Kisler K Nelson AR Montagne A Zlokovic BV. Cerebral blood flow regulation and neurovascular dysfunction in Alzheimer disease. Nat Rev Neurosci. (2017) 18:419–34. 10.1038/nrn.2017.48 28515434 PMC5759779

[334] Bordeleau M ElAli A Rivest S. Severe chronic cerebral hypoperfusion induces microglial dysfunction leading to memory loss in APPswe/PS1 mice. Oncotarget. (2016) 7:11864–80. 10.18632/oncotarget.7689 26918610 PMC4914254

[335] D'haeseleer M Hostenbach S Peeters I Sankari SE Nagels G De Keyser J. Cerebral hypoperfusion: a new pathophysiologic concept in multiple sclerosis? J Cereb Blood Flow Metab. (2015) 35:1406–10. 10.1038/jcbfm.2015.131 26104292 PMC4640326

[336] Keymeulen B Jacobs A de Metz K de Sadeleer C Bossuyt A Somers G. Regional cerebral hypoperfusion in long-term type 1 (insulin-dependent) diabetic patients: relation to hypoglycaemic events. Nucl Med Commun. (1995) 16:10–6. 10.1097/00006231-199501000-00004 7609929

[337] Love S Miners JS. Cerebral hypoperfusion and the energy deficit in Alzheimer's disease. Brain Pathol. (2016) 26:607–17. 10.1111/bpa.12401 27327656 PMC8028913

[338] Iadecola C. The neurovascular unit coming of age: a journey through neurovascular coupling in health and disease. Neuron. (2017) 96:17–42. 10.1016/j.neuron.2017.07.030 28957666 PMC5657612

[339] Xanthos DN Sandkühler J. Neurogenic neuroinflammation: inflammatory CNS reactions in response to neuronal activity. Nat Rev Neurosci. (2014) 15:43–53. 10.1038/nrn3617 24281245

[340] Maher CO Anderson RE Martin HS McClelland RL Meyer FB. Interleukin-1beta and adverse effects on cerebral blood flow during long-term global hypoperfusion. J Neurosurg. (2003) 99:907–12. 10.3171/jns.2003.99.5.0907 14609172

[341] Farkas E Süle Z Tóth-Szuki V Mátyás A Antal P Farkas IG. Tumor necrosis factor-alpha increases cerebral blood flow and ultrastructural capillary damage through the release of nitric oxide in the rat brain. Microvasc Res. (2006) 72:113–9. 10.1016/j.mvr.2006.05.007 16854437

[342] Sibson NR Blamire AM Perry VH Gauldie J Styles P Anthony DC. TNF-alpha reduces cerebral blood volume and disrupts tissue homeostasis via an endothelin- and TNFR2-dependent pathway. Brain. (2002) 125(Pt 11):2446–59. 10.1093/brain/awf256 12390971

[343] Tureen J. Effect of recombinant human tumor necrosis factor-alpha on cerebral oxygen uptake, cerebrospinal fluid lactate, and cerebral blood flow in the rabbit: role of nitric oxide. J Clin Invest. (1995) 95:1086–91. 10.1172/JCI117755 7883956 PMC441444

[344] Moore CI Cao R. The hemo-neural hypothesis: on the role of blood flow in information processing. J Neurophysiol. (2008) 99:2035–47. 10.1152/jn.01366.2006 17913979 PMC3655718

[345] Sweeney MD Ayyadurai S Zlokovic BV. Pericytes of the neurovascular unit: key functions and signaling pathways. Nat Neurosci. (2016) 19:771–83. 10.1038/nn.4288 27227366 PMC5745011

[346] McConnell HL Kersch CN Woltjer RL Neuwelt EA. The translational significance of the neurovascular unit. J Biol Chem. (2017) 292:762–70. 10.1074/jbc.R116.760215 27920202 PMC5247651

[347] Carmichael MD Davis JM Murphy EA Carson JA Van Rooijen N Mayer E. Role of brain macrophages on IL-1beta and fatigue following eccentric exercise-induced muscle damage. Brain Behav Immun. (2010) 24:564–8. 10.1016/j.bbi.2009.12.011 20051263

[348] Hladky SB Barrand MA. Mechanisms of fluid movement into, through and out of the brain: evaluation of the evidence. Fluids Barriers CNS. (2014) 11:26. 10.1186/2045-8118-11-26 25678956 PMC4326185

[349] Fantini S Sassaroli A Tgavalekos KT Kornbluth J. Cerebral blood flow and autoregulation: current measurement techniques and prospects for noninvasive optical methods. Neurophotonics. (2016) 3:031411. 10.1117/1.NPh.3.3.031411 27403447 PMC4914489

[350] Pollock JD Makaryus AN Physiology, Cardiovascular Hemodynamics (2019). 29261894

[351] Castro P Azevedo E Sorond F. Cerebral autoregulation in stroke. Curr Atheroscler Rep. (2018) 20:37. 10.1007/s11883-018-0739-5 29785667

[352] Mösges R Kaatz V Schmalz P Meiser P Eschmann K. Glycerol lidocaine eardrops for the treatment of acute abacterial otitis externa. Arzneimittelforschung. (2010) 60:427–31. 10.1055/s-0031-1296307 20712132

[353] Marrie RA Yu BN Leung S Elliott L Caetano P Warren S. Rising prevalence of vascular comorbidities in multiple sclerosis: validation of administrative definitions for diabetes, hypertension, and hyperlipidemia. Mult Scler. (2012) 18:1310–9. 10.1177/1352458512437814 22328682

[354] Guzik TJ Touyz RM. Oxidative stress, inflammation, and vascular aging in hypertension. Hypertens. (2017) 70:660–7. 10.1161/HYPERTENSIONAHA.117.07802 28784646

[355] Quintana FJ. Astrocytes to the rescue! Glia limitans astrocytic endfeet control CNS inflammation. J Clin Invest. (2017) 127:2897–9. 10.1172/JCI95769 28737511 PMC5531401

[356] Park S Lakatta EG. Role of inflammation in the pathogenesis of arterial stiffness. Yonsei Med J. (2012) 53:258–61. 10.3349/ymj.2012.53.2.258 22318811 PMC3282971

[357] Thenappan T Prins KW Pritzker MR Scandurra J Volmers K Weir EK. The critical role of pulmonary arterial compliance in pulmonary hypertension. Ann Am Thorac Soc. (2016) 13:276–84. 10.1513/AnnalsATS.201509-599FR 26848601 PMC5461956

[358] Barnes JN Nualnim N Sugawara J Sommerlad SM Renzi CP Tanaka H. Arterial stiffening, wave reflection, and inflammation in habitually exercising systemic lupus erythematosus patients. Am J Hypertens. (2011) 24:1194–200. 10.1038/ajh.2011.143 21833040

[359] Tryfonopoulos D Anastasiou E Protogerou A Papaioannou T Lily K Dagre A. Arterial stiffness in type 1 diabetes mellitus is aggravated by autoimmune thyroid disease. J Endocrinol Invest. (2005) 28:616–22. 10.1007/BF03347260 16218044

[360] Fjeldstad C Frederiksen C Fjeldstad AS Bemben M Pardo G. Arterial compliance in multiple sclerosis: a pilot study. Angiology. (2010) 61:31–6. 10.1177/0003319709334120 19497928

[361] Cojocaru M Cojocaru IM Silosi I Vrabie CD. Pulmonary manifestations of systemic autoimmune diseases. Maedica. (2011) 6:224–9. 22368703 PMC3282547

[362] Moldoveanu B Otmishi P Jani P Walker J Sarmiento X Guardiola J. Inflammatory mechanisms in the lung. J Inflamm Res. (2009) 2:1–11. 22096348 PMC3218724

[363] Losa García JE Rodríguez FM Martín de Cabo MR García Salgado MJ Losada JP Villarón LG Evaluation of inflammatory cytokine secretion by human alveolar macrophages. Mediators Inflamm. (1999) 8:43–51. 10.1080/09629359990711 10704089 PMC1781780

[364] Oliveira RKF Agarwal M Tracy JA Karin AL Opotowsky AR Waxman AB. Age-related upper limits of normal for maximum upright exercise pulmonary haemodynamics. Eur Respir J. (2016) 47:1179–88. 10.1183/13993003.01307-2015 26677941

[365] Bazelmans E Bleijenberg G Vercoulen JH van der Meer JW Folgering H. The chronic fatigue syndrome and hyperventilation. J Psychosom Res. (1997) 43:371–7. 10.1016/S0022-3999(97)00169-4 9330236

[366] Stewart JM Medow MS Cherniack NS Natelson BH. Postural hypocapnic hyperventilation is associated with enhanced peripheral vasoconstriction in postural tachycardia syndrome with normal supine blood flow. Am J Physiol Heart Circ Physiol. (2006) 291:H904–13. 10.1152/ajpheart.01359.2005 16565300 PMC4511478

[367] Coelho R Hughes AM da Fonseca AF Bond MR. Essential hypertension: the relationship of psychological factors to the severity of hypertension. J Psychosom Res. (1989) 33:187–96. 10.1016/0022-3999(89)90046-9 2724195

[368] Shanks L Jason LA Evans M Brown A. Cognitive impairments associated with CFS and POTS. Front Physiol. (2013) 4:113. 10.3389/fphys.2013.00113 23720636 PMC3655280

[369] Depiazzi J Everard ML. Dysfunctional breathing and reaching one's physiological limit as causes of exercise-induced dyspnoea. Breathe. (2016) 12:120–9. 10.1183/20734735.007216 27408630 PMC4933621

[370] Földi M Csillik B Zoltán OT. Lymphatic drainage of the brain. Experientia. (1968) 24:1283–7. 10.1007/BF02146675 5703044

[371] Xie L Kang H Xu Q Chen MJ Liao Y Thiyagarajan M. Sleep drives metabolite clearance from the adult brain. Science. (2013) 342:373–7. 10.1126/science.1241224 24136970 PMC3880190

[372] Schiweck J Eickholt BJ Murk K. Important shapeshifter: mechanisms allowing astrocytes to respond to the changing nervous system during development, injury and disease. Front Cell Neurosci. (2018) 12:261. 10.3389/fncel.2018.00261 30186118 PMC6111612

[373] Ngo ST Steyn FJ McCombe PA. Gender differences in autoimmune disease. Front Neuroendocrinol. (2014) 35:347–69. 10.1016/j.yfrne.2014.04.004 24793874

[374] Cade BE Chen H Stilp AM Gleason KJ Sofer T Ancoli-Israel S. Genetic associations with obstructive sleep apnea traits in hispanic/latino Americans. Am J Respir Crit Care Med. (2016) 194:886–97. 10.1164/rccm.201512-2431OC 26977737 PMC5074655

[375] Wang T Yin J Miller AH Xiao C. A systematic review of the association between fatigue and genetic polymorphisms. Brain Behav Immun. (2017) 62:230–44. 10.1016/j.bbi.2017.01.007 28089639 PMC5947855

